# Entropy generation and regression analysis of unsteady Carreau ternary hybrid nanofluid flow with electromagnetic and thermal influences

**DOI:** 10.1186/s11671-025-04408-2

**Published:** 2025-12-15

**Authors:** T. Sindhu, K. Jagadeeshkumar

**Affiliations:** Department of Mathematics, School of Advanced Sciences, Vellore Institute of Technology, Vellore, Tamil Nadu 632014 India

**Keywords:** Carreau fluid, Ternary hybrid nanofluid, Electromagnetic effects, Entropy generation, Thermal radiation, Regression analysis

## Abstract

This work examines the transient flow and heat transfer characteristics of a Carreau ternary hybrid nanofluid ($\hbox {TiO}_2$ + Cu + $\hbox {Al}_2\hbox {O}_3$/CMC–$\hbox {H}_2$O) across a stretched sheet subjected to combined electromagnetic and thermal influences. The model integrates suction/injection, heat radiation, Ohmic dissipation, and concentration fluctuations, emphasising entropy generation. The governing nonlinear equations are converted using similarity variables and solved numerically using the BVP5c technique. Multiple linear regression is used to forecast skin friction, Nusselt number, and Sherwood number. Results indicate that magnetic and electric field intensities, Weissenberg number, and thermal radiation substantially affect velocity, temperature, and concentration distributions, but entropy production underscores irreversibility processes. The ternary hybrid nanofluid has enhanced thermal performance relative to mono and binary nanofluids, presenting potential advantages for cooling, extrusion, and coating applications.

Due of their many technical and industrial uses, non-Newtonian fluids have been studied. These fluids are essential to paint manufacture, blood flow analysis, paper production, plastic sheet creation, cosmetics, polymeric fluids, oil drilling, glass shaping, biological solutions, and adhesives. Non-Newtonian nanofluids are created by mixing nanoparticles with base fluid. Hybrid nanofluids with two kinds of nanoparticles improve their thermal and rheological characteristics. This upgrade makes them suitable for heat exchangers, cooling systems, and solar energy. The most sophisticated nanofluid has three nanoparticles: ternary hybrid. These optimise stability, viscosity, and thermal performance. Biomedical gadgets and renewable energy systems use ternary hybrid nanofluids, advancing technology and energy efficiency.

In 1995, Choi presented the concept of nanofluids [1]. arguing that distributing nanoparticles in a base fluid can considerably boost its thermal conductivity. Their findings revealed significant improvements in heat conductivity when nanoparticles were added into fluids such as water. Bi et al. [2] investigates $TiO_{2}$ nanoparticles in mineral oil enhance the functionality and energy efficiency of HFC134a home freezers, according to the study’s experimental findings. Carreau [3] initially proposed the Carreau model in 1972. The text outlines the flow behaviour of non-Newtonian fluids under both low and high shear rates. Khan and Hashima investigated nonlinear stretching sheets in Carreau fluids [4]. Tian et al. [5] explain how Prandtl ternary hybrid nanofluid flows around a heated cylinder. They show that Hall and ion slip reduce entropy. In contrast, Brinkman and magnetic effects increase irreversibility. The Keller-Box technique is used by Zafar et al. [6] to explain the mixed convective flow of Carreau fluids including gyrotactic microorganisms over a Riga sheet. Dual diffusion, nanoparticle control, and entropy-related aspects are examined. Using BVP4C to investigate heat, mass transport, and motile density, Zafar et al. [7] clarify Prandtl nanofluid flow including gyrotactic microorganisms over a stretchy cylinder under Darcy–Forchheimer effects.

Magnetohydrodynamics (MHD) investigates the dynamics of electrically conductive fluids inside magnetic fields, with much research dedicated to the heat and mass transport of nanofluids. Bachok et al. [8] documented dual solutions for thermal transport over elastic and shrinking surfaces, while Shah et al. [9] used the Buongiorno model to investigate MHD micropolar nanofluid bioconvection including motile microorganisms. Nandi et al. [10] highlighted the complexities of heat transmission in nanofluids influenced by thermal radiation, emphasising their industrial significance. Rehman et al. [11] explain Darcy-Forchheimer nanofluid bioconvection with slip effects. They show that thermophoresis increases nanoparticle concentration. At the same time, higher Darcy and Prandtl numbers reduce velocity and temperature profiles. Rehman et al. [12] investigated Eyring–Powell nanofluid blood flow, while Kaswan et al. [13] and Kumar et al. [14] studied hybrid and Casson nanofluids, emphasising skin friction, entropy production, and heat transfer irreversibilities. Agrawal et al. [15] and Kumar et al. [16] investigated hybrid nanofluids incorporating thermal radiation and porous media, documenting improved heat and mass transfer.Faizan et al. [17] examined Walter-B nanofluids containing motile microorganisms, Shah et al. [18] utilised the Laplace transform for unsteady hybrid nanofluid flow analysis, and Jubair et al. [19] investigated Casson hybrid nanofluids integrated with phase change material (PCM), addressing the influences of magnetic fields, Joule heating, and thermophoresis.

Thermal radiation is the energy that a material gives off because of its internal energy, and the amount of energy it gives off depends on the temperature. Using the Keller-Box approach, Shahzad et al. [20] investigated heat transfer and Casson hybrid nanofluid flow in parabolic trough solar collectors on aeroplane wings, showing increases in thermal efficiency of up to 21.8. Rehman et al. [21] explain Jeffery-Hamel non-Newtonian flow in nonparallel channels by applying non-Fourier’s law. They show that thermal relaxation lowers temperature, while thermophoresis boosts heat and concentration fluxes. Ouni et al. [22] investigated hybrid nanofluid flow over solar collectors using the Oldroyd-B model, and Narahari explored radiation and natural convection between parallel plates. Mkhastwa et al. [23] examined electromagnetic Carreau hybrid nanofluid flow in porous media, whereas Nandy et al. [10] examined solution multiplicity and stability. Johnson et al. [24], thermal buoyancy improves cooling and Walters’ B fluid flow. In their study of Casson, Maxwell, and Williamson nanofluids on porous and stretched surfaces in parabolic trough solar collectors, Jamshed et al. [25–27] reported thermal efficiency improvements of 1.5–29, depending on the fluids and nanoparticles. In their analysis of hybrid nanofluid and Prandtl-Eyring fluid flows in coaxial cylinders and on curved Riga surfaces under thermal radiation, heat sources, viscous dissipation, and PCM effects, Ali et al. [28, 29] focused on the effects of temperature, slip, entropy, and velocity. Ag–$\hbox {Fe}_3\hbox {O}_4$ hybrid nanofluid flow across inclined permeable surfaces was examined by Jubair et al. [30], taking into account mixed convection, chemical reactions, permeability, and heat transfer performance.

Electromagnetic forces in electrically conducting fluids offer an effective means of controlling flow separation. Khedher et al. [31] explain the flow of Carreau nanofluid in an inclined porous channel. They show that Darcy and Forchheimer effects increase entropy production. At the same time, porosity reduces the Bejan number and skin friction. Chakraborty et al. [32] conduct an analytical study of thermally fully developed electromagnetohydrodynamic (EMHD) flows in narrow conduits with constant wall heat flux, considering both electromagnetic and electrokinetic processes. The study highlights substantial effects on temperature profiles, velocity, and Nusselt number, elucidating the complexities of micro/nanoscale thermal management systems. Rehman et al. [33] utilise the Cattaneo-Christov model to analyse the wedge flow of $\hbox {CoFe}_2\hbox {O}_4$/water nanoliquid, focussing on suction effects, thermal relaxation, nanoparticle volume, and the distinction between stable and unstable flow solutions. Alkathiri et al. [34] employ Galerkin FEM to analyse the effects of magnetism and viscosity, elucidating heat transfer and entropy formation in the flow of binary nanofluids over a parabolic trough solar collector. Ali et al. [35] examine heat and mass transfer, velocity, and microorganism dynamics to characterise the bioconvective flow of Sutterby nanofluid on a rotating disc, employing the Keller-Box method alongside the Cattaneo-Christov model.

An irreversible system and its surroundings are measured by entropy, which measures heat and matter dissipation. Higher entropy generation frequently means thermal systems operate less efficiently. Recent research has improved electric heater and nanofluid flow performance. According to Nandi et al. [36], Weissenberg and Brinkmann numbers greatly impact entropy and velocity in Carreau ternary hybrid nanofluid flows, taking into account aspects such as slip and heat transmission. Makhdoum et al. [37] examined the effects of heat production and nanoparticle clustering on entropy, while Rafique et al. [38] found that oblate spheroidal nanoparticles increase thermal entropy in $Al_2O_3H_2O$ flows. Entropy generation in radiative flow of a nanoliquid under a rotating frame with activation energy, velocity slip, and porous medium effects is studied using Buongiorno’s model and bvp4c. Maiti et al. [39] discovered that nanoparticle concentration impacts hybrid nanofluid flow, temperature, and entropy under magnetic fields and thermal radiation. Boujelbene et al. [40] explain that magnetic and Brinkman parameters enhance entropy, while heat source and thermal stratification have opposite effects on temperature in Prandtl nanofluid flow over a cylinder. AlAbdulaal et al. [41] explain entropy generation and heat transfer in Jeffrey-Hamel biviscosity Bingham nanofluid flow with Marangoni convection, highlighting effects on velocity, temperature, drag, and system performance. Shahzad et al. [42] explain hybrid nanofluid flow and heat transfer with entropy generation over a parabolic trough solar collector using Oldroyd-B model and Keller-Box scheme. Jamshed et al. [43]explain entropy generation and heat transfer in Maxwell nanofluid flow over parabolic trough solar collectors using Keller-Box method, comparing Cu-EO and $\hbox {ZrO}_2$-EO performance. Arafat et al. [44] explain Darcy–Forchheimer bioconvective nanoliquid flow over a porous disk using Buongiorno’s model and bvp4c, analyzing entropy generation, velocity, concentration, and microbial dynamics.

The relationship between the governing physical factors and key output parameters–skin friction, Nusselt number, and Sherwood number–is statistically modelled through multiple linear regression analysis. Shaped $\hbox {Al}_2\hbox {O}_3$ nanoparticles in nanofluids enhance heat transfer, thereby improving thermal management in electronics, biomedical devices, solar panels, and various industrial applications, as noted by Priyadharshini et al. [45]. Fisal Asiri et al. [46] discuss how mixed convection and Buongiorno effects enhance heat transfer, whereas thermal and solutal relaxation diminish heat and mass transfer in the flow of ferromagnetic tangent hyperbolic nanofluids. SWCNT-MWCNT hybrid nanofluids improve heat transport in porous media influenced by magnetohydrodynamic and thermal effects, thereby aiding in advanced thermal system designs, as noted by Blessy et al. [47]. Ayub et al. [48] demonstrate that cross nanofluid heat transfer in porous media, under conditions of mixed convection and magnetic fields, is significantly affected by second-order slip and thermophoretic effects. Alimi et al. [49] investigate heat transfer in water-based hybrid nanofluid flow over a stretching surface, employing the ANN and Cattaneo–Christov model to analyse the effects of magnetic fields, thermal conditions, and nanoparticles. Jubair et al. [50] analyse wastewater discharge through Walter’s B and second-grade nanofluids on a Riga surface utilising ANN-LMBOA, with results validated by bvp4c and statistical accuracy measures.

- The current issue has immediate technical implications in polymer sheet extrusion, cooling electronic components, thermal management in microfluidic devices, and metallurgical processes using electrically conductive non-Newtonian nanofluids.
- The model applies to today’s energy and material processing sectors since it takes into account the impacts of electromagnetic fields, heat radiation, and ternary nanoparticles.

To guide the present investigation, the following research questions are posed:

1. How do electromagnetic forces, thermal radiation, and unsteadiness influence the velocity, temperature, concentration, and entropy generation in Carreau ternary hybrid nanofluid flow?
2. In what way does the combination of $\hbox {TiO}_2$, Cu, and $\hbox {Al}_2\hbox {O}_3$ nanoparticles enhance heat and mass transfer performance compared with mono and binary nanofluids?
3. Can multiple linear regression (MLR) provide accurate and generalizable predictions for key engineering parameters such as skin friction, Nusselt number, and Sherwood number?
4. What are the practical implications of the obtained results for advanced thermal systems such as cooling devices, extrusion processes, and coating technologies?

**Novelty**

Although numerous studies have investigated non-Newtonian hybrid nanofluids and their thermal transport characteristics, the transient behaviour of Carreau ternary hybrid nanofluids influenced by coupled electromagnetic hydrodynamic (EMHD) and entropy factors has not been documented. This study represents the first investigation of a $\hbox {TiO}_2$ + Cu + $\hbox {Al}_2\hbox {O}_3$/CMC–$\hbox {H}_2$O ternary hybrid nanofluid moving over a stretched sheet, affected simultaneously by suction/injection, Lorentz forces, Ohmic dissipation, thermal radiation, and entropy production. The selection of $\hbox {TiO}_2$, Cu, and $\hbox {Al}_2\hbox {O}_3$ nanoparticles is informed by their complementary thermophysical properties: copper exhibits high thermal conductivity, $\hbox {Al}_2\hbox {O}_3$ demonstrates chemical stability and durability, and $\hbox {TiO}_2$ enhances photocatalytic and thermal performance. The incorporation of these elements into a CMC–$\hbox {H}_2$O base fluid yields a synergistic effect, enhancing both heat transfer and rheological stability. This phenomenon has not been previously investigated in the context of Carreau-type non-Newtonian fluids. The research utilises similarity transformations and the BVP5c solver to obtain numerically stable solutions, while employing multiple linear regression (MLR) to predict and generalise key engineering responses, such as skin friction, Nusselt number, and Sherwood number. In this context, MLR is innovative as it provides a statistical framework for assessing parameter sensitivity and identifying predictive relationships applicable in engineering design, thereby eliminating the necessity for full-scale numerical simulations on each occasion. This study offers a significant contribution to the modelling and optimisation of ternary hybrid nanofluids for practical applications such as cooling, extrusion, and thermal coating technologies. It integrates advanced rheology, customised nanoparticle selection, entropy analysis, and regression-based prediction.

## Mathematical formulation

**Table 1:** Thermophysical properties of (*CMC*-water), *Cu*, $(Al_{2}O_{3})$, $(TiO_{2})$

Physical properties	$\rho$ (kg/m^3^)	*k* (W/mK)	$C_{p}$ (J/KgK)	$\sigma$ (S/m)
$Al_{2}O_{3}$	3970	40	765	3.69 × 10^7^
*Cu*	8933	401	385	5.96 × 10^7^
$TiO_{2}$	4250	8.95	686.2	2.38 × 10^6^
(*CMC*-water)	997.1	0.613	4179	0.05

This investigation examines the laminar, incompressible flow of a Carreau ternary hybrid nanofluid across a linearly elongating surface. The nanofluid consists of copper (Cu), aluminium oxide ($Al_{2}O_{3}$), and titanium dioxide ($TiO_{2}$) nanoparticles suspended in carboxymethylcellulose (CMC) water, which was selected for its improved rheological and thermal characteristics. Thermal radiation, ohmic dissipation, an applied magnetic field, a temperature jump condition, heat generation, and time-dependent changes in particle concentration are some of the effects that are included in this configuration. The electromagnetic hydrodynamic (EMHD) impact also affects the flow; it produces Lorentz forces that alter the electrically conductive fluid’s behaviour through the interplay of magnetic and electric fields. By using its $\tilde{x}$ and $\tilde{y}$ axes, the velocity components $\tilde{u}$ and $\tilde{v}$ are assessed in the coordinate system. The following boundary conditions define the system: $\tilde{U}_{wall}=\frac{a_{0}\tilde{x}}{1-a\tilde{t}}$ provides the fluid velocity on the wall, where $a_{0}$ is a positive constant and adds a temporal dependency that allows the stretching surface to change dynamically over time. The surface temperature is determined by the formula $\tilde{T}_{wall}=\tilde{T}_{\infty }+\frac{b_{0}\tilde{x}}{1-a\tilde{t}}$, where $b_{0}$ establishes the temperature gradient and $\tilde{T}_{\infty }$ is the temperature far from the field. The concentration at the wall is determined by the formula $\tilde{C}_{wall}= \tilde{C}_{\infty }+\frac{c_{0}\tilde{x}}{1-a\tilde{t}}$, where $c_{0}$ establishes the concentration gradient and $\tilde{C_{\infty }}$ is the concentration far from the field. $B=\frac{B_{0}}{\sqrt{1-a\tilde{t}}}$ represents the magnetic field, and $E=\frac{E_{0}}{\sqrt{1-a\tilde{t}}}$ represents the electric field applied to the flow.

**Fig. 1:**
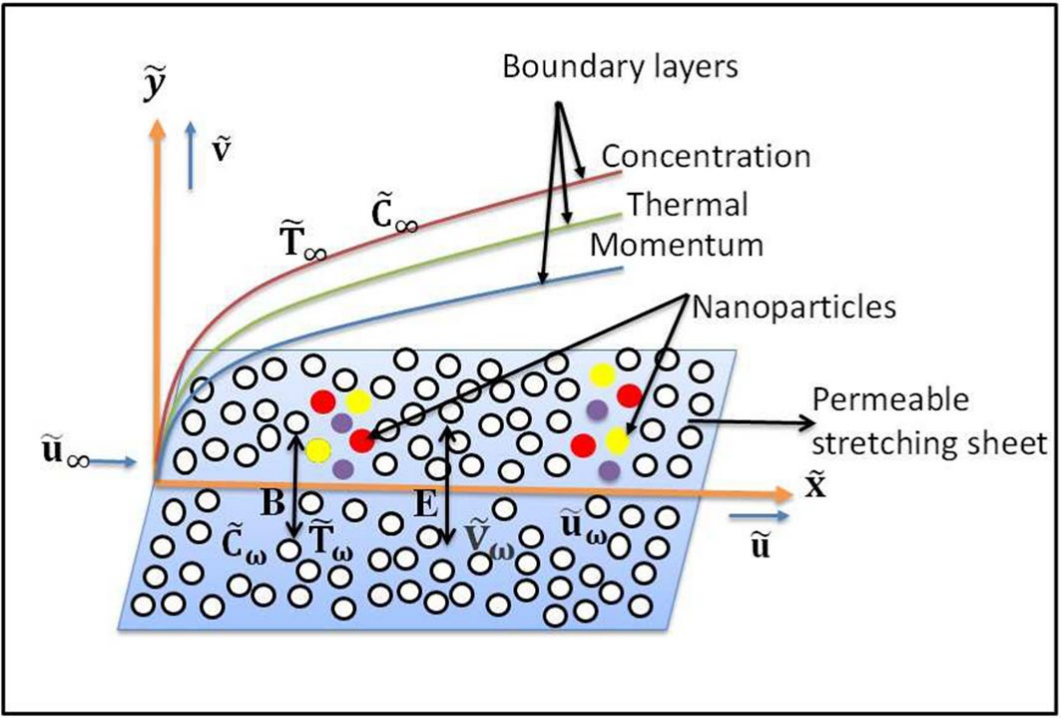
Physical configuration

This arrangement provides a basis for investigating the heat and mass transmission characteristics of the tri-hybrid nanofluid flow, together with its time-dependent thermal and concentration profiles. The constitutive equation is expressed by $\tau = -pI + \mu (\dot{\gamma }) A_{1}$, where $\mu$ denotes the apparent viscosity, $p$ stands for pressure, $I$ denotes the identity tensor, and $A_{1}$ is the first Rivlin-Ericksen tensor, given by $A_{1} = (\nabla \vec {V}) + (\nabla \vec {V})^{T}$. The apparent viscosity $\mu$ is expressed as

1 $$\begin{aligned} \tilde{\mu } = \tilde{\mu }_{\infty } \left\{ 1 + \left( \frac{\tilde{\mu }_{0} - \tilde{\mu }_{\infty }}{\tilde{\mu }_{\infty }}\right) \left[ 1 + (\Gamma \dot{\gamma })^2\right] ^{\frac{n-1}{2}} \right\} \end{aligned}$$

in which $\tilde{\mu }_{0}$ denotes zero shear rate of the viscosity, while $\tilde{\mu }_{\infty }$ represents the infinite shear rate of the viscosity, $\dot{\gamma }$ is the shear rate, $n$ is the power-law index, and $\Gamma$ is the material parameter.The shear rate $\dot{\gamma }$ is defined as $\dot{\gamma } = \frac{\sqrt{A_{1}^2}}{\sqrt{2}}$, indicating the correlation between strain rate and viscosity in the fluid model. The following nonlinear governing equations for the Carreau ternary hybrid nanofluid may be obtained by considering the work of [36, 51–53] (Fig. 1).

2 $$\begin{aligned} & \frac{\partial \tilde{u}}{\partial \tilde{x}}+\frac{\partial \tilde{v}}{\partial \tilde{y}} = 0, \end{aligned}$$

3 $$\begin{aligned} & \begin{aligned} \frac{\partial \tilde{u}}{\partial \tilde{t}}+\tilde{u}\frac{\partial \tilde{u}}{\partial \tilde{x}}+\tilde{v}\frac{\partial \tilde{u}}{\partial \tilde{y}}&= \frac{\mu _{thnf}}{\rho _{thnf}}\frac{\partial ^{2}\tilde{u}}{\partial \tilde{y}^{2}}\left\{ 1+\Gamma ^{2}\left( \frac{\partial \tilde{u}}{\partial \tilde{y}}\right) ^{2}\right\} ^\frac{n-1}{2} \\&\quad + \frac{\mu _{thnf}}{\rho _{thnf}}(n-1)\Gamma ^{2}\frac{\partial ^{2}\tilde{u}}{\partial \tilde{y}^{2}}\left( {\frac{\partial \tilde{u}}{\partial \tilde{y}}}\right) ^{2}\left\{ 1+\Gamma ^{2}\left( \frac{\partial \tilde{u}}{\partial \tilde{y}}\right) ^{2}\right\} ^\frac{n-3}{2} \\&\quad -\frac{\sigma _{thnf}}{\rho _{thnf}}\left( EB-B^{2}\tilde{u}\right) \end{aligned} \end{aligned}$$

4 $$\begin{aligned} & \begin{aligned} (\rho cp)_{thnf}\left( \tilde{v}\frac{\partial \tilde{T}}{\partial \tilde{y}}+\tilde{u}\frac{\partial \tilde{T}}{\partial \tilde{x}}+\frac{\partial \tilde{T}}{\partial \tilde{t}}\right)&=k_{thnf} \frac{\partial ^{2}\tilde{T}}{\partial \tilde{y}^{2}} - \frac{\partial q_{r}}{\partial \tilde{y}} \\&\quad + \sigma _{thnf} \left( \tilde{u}B - E\right) ^{2} +{Q_0}(\tilde{T}-\tilde{T_{\infty }}) \end{aligned} \end{aligned}$$

5 $$\begin{aligned} & \begin{aligned} \tilde{v}\frac{\partial \tilde{C}}{\partial \tilde{y}}+\tilde{u}\frac{\partial \tilde{C}}{\partial \tilde{x}}+\frac{\partial \tilde{C}}{\partial \tilde{t}}&= D_{\text {thnf}}\frac{\partial ^{2}\tilde{C}}{\partial \tilde{y}^{2}}+ \text {K}(\tilde{C}-\tilde{C_{\infty }}) \end{aligned} \end{aligned}$$

The pertinent boundary conditions are [51, 54]:

6 $$\begin{aligned} \begin{aligned}&\tilde{u} = \tilde{u}_w = \frac{a_{0}x}{1-a\tilde{t}}, \; \tilde{v} = -\tilde{v}_w, \; -k_{thnf}\frac{\partial \tilde{T}}{\partial \tilde{y}} = h_{f}(\tilde{T}_w - \tilde{T}), \; \tilde{C} = \tilde{C}_w \; \text {at} \; \tilde{y} = 0, \\&\tilde{u} \rightarrow 0, \; \tilde{T} \rightarrow \tilde{T}_{\infty }, \; \tilde{C} \rightarrow \tilde{C}_{\infty } \; \text {as} \; \tilde{y} \rightarrow \infty . \end{aligned} \end{aligned}$$

The continuity equation, which guarantees mass conservation in the flow, is given by Eq. (2). The Navier–Stokes equations are used to develop the momentum equation, Eq. (3), which takes into account the implications of the Carreau model for non-Newtonian fluids. The left-hand side of Eq. (3) accounts for advective and unstable components, whereas the first two terms on the right-hand side explain the shear-dependent viscosity and elastic behaviour of the Carreau nanofluid. The influence of the electromagnetic field, including contributions from the applied magnetic field and electric field via Lorentz forces, is captured by the third component in Eq. (3). The effects of porosity on the permeable medium are examined in the fourth semester. The system’s heat transmission methods are described by the energy equation, Eq. (4). The right-side beginning word denotes heat conduction because of the thermal conductivity of the nanofluid. In order to account for radiative heat flow in the system, the second term incorporates the effect of thermal radiation via $q_r$. The following is the expression for the radiative heat flow $q_r$ [10]:

7 $$\begin{aligned} q_r = -\frac{4\tilde{\sigma }}{3\tilde{k}} \frac{\partial \tilde{T}^4}{\partial \tilde{y}}, \end{aligned}$$

where $\tilde{\sigma }$ signifies the Stefan-Boltzmann constant and $\tilde{k}$ indicates the mean absorption coefficient. Third term of Eq. (4) considers Ohmic dissipation resulting from the electromagnetic field, while the fourth term accounts for heat generation or absorption within the system. Advection, mass diffusion, and the effects of chemical reactions are all included in the concentration dynamics model, which is provided by Eq. (5).The term $K(\tilde{C} - \tilde{C}_{\infty })$ refers to reactive diffusion in the boundary layer. It models the interactions between nanoparticles and fluid or surface reactions that happen during coating and cooling processes. Although the flow is described as stagnation-type, reactive mass transfer is included for physical completeness. These equations, along with their accompanying boundary conditions, form a comprehensive mathematical framework for analyzing the behavior of the Carreau ternary hybrid nanofluid in unsteady, electromagnetically affected situations.

The boundary conditions at the surface ($\tilde{y} = 0$) are delineated by Eq. (6). The velocity $\tilde{u}$ at the wall is expressed as $\frac{a_{0}x}{1 - a\tilde{t}}$, with $\tilde{t}$ representing the time-dependent parameter and $a_{0}$ being a constant linked to the stretching rate. This stretching generates fluid motion in the boundary layer. The surface temperature gradient is expressed as $-\text {k}_{thnf} \frac{\partial \tilde{T}}{\partial \tilde{y}} = h_{f}(\tilde{T}_{f} - \tilde{T})$, representing heat transport through a convective boundary condition. At the boundary, the concentration $\tilde{C}$ is established as $\tilde{C}_{w}$.

The conditions at the boundary in the area far from the surface are defined by Eq. (6). As $\tilde{y}$ increases indefinitely, the velocity tends towards zero ($\tilde{u} \rightarrow 0$), the temperature aligns with the ambient temperature ($\tilde{T} \rightarrow \tilde{T}_{\infty }$), and the concentration approaches the ambient concentration ($\tilde{C} \rightarrow \tilde{C}_{\infty }$).

Similarity transformations make it simpler to solve problems by simplifying governing equations.

8 $$\begin{aligned} \begin{aligned} \psi&= \sqrt{\frac{ \nu _{f}\cdot a_0}{1 - a\tilde{t}}} \tilde{x} f(\eta ), \quad \frac{\eta }{\tilde{y}} = \sqrt{\frac{a_0}{\nu _f(1 - a\tilde{t})}}, \quad \tilde{u} = \frac{a_0}{1 - a\tilde{t}} \tilde{x} f'(\eta ), \\ \tilde{v}&= -\sqrt{\frac{a_0 \nu _f}{1 - a\tilde{t}}} f(\eta ), \quad \theta (\eta ) = \frac{\tilde{T} - \tilde{T}_{\infty }}{\tilde{T}_w - \tilde{T}_{\infty }}, \quad \phi (\eta ) = \frac{\tilde{C} - \tilde{C}_{\infty }}{\tilde{C}_w - \tilde{C}_{\infty }} \end{aligned} \end{aligned}$$

where $f^{'}$ represents primary velocity and $\eta$ denotes the similarity parameter.

The similarity transformation used in this study, where *y* [m] is the normal coordinate and $\nu$ [$\hbox {m}^2$/s] is the kinematic viscosity. Since *y* has units of length [m] and $\sqrt{a_0/\nu _f}$ has units of [$\hbox {m}^{-1}$], the similarity variable $\eta$ is dimensionless. This guarantees that the transformed governing equations and their boundary conditions are nondimensional and dimensionally consistent. Using the similarity transformation provided in Eq. (8), Eqs. (3), (4), and (5) were converted into nonlinear ordinary differential equations. The following are the updated equations that resulted:

9 $$\begin{aligned} \begin{aligned}&\frac{A_1}{A_2}f'''\left[ \left( 1+We^2 f''^2\right) ^{\frac{n-1}{2}} + (n-1) We^2 f''^2\left( 1+We^2 f''^2\right) ^{\frac{n-3}{2}}\right] \\&\quad - \frac{A_3 A_4 A_5}{A_2}M\left( E_1 - f'\right) - \delta \left( f''\frac{\eta }{2}+f'\right) - f'^2 + f f'' = 0. \end{aligned} \end{aligned}$$

10 $$\begin{aligned} \begin{aligned}&\theta ^{''}\left[ A_{6}A_{7}A_{8} + \frac{4}{3}Rd\right] + A_{3}A_{4}A_{5}MEcPr\left( f^{'} - E_1\right) ^{2} + QPr\theta \\&\quad - A_{9}Pr\delta \left( \theta ^{'}\frac{\eta }{2} + \theta \right) - A_{9}Prf^{'}\theta + A_{9}Prf\theta ^{'} = 0, \end{aligned} \end{aligned}$$

11 $$\begin{aligned} \begin{aligned} A_{10}\phi ^{''}+ K_{r} Sc\phi -\delta Sc\left[ \phi ^{'}\frac{\eta }{2}+\phi \right] -Sc f^{'}\phi +Sc f \phi ^{'}=0, \end{aligned} \end{aligned}$$

where $A_{1}=\frac{\mu _{thnf}}{\mu _{f}}, \mu _{f}$ represents the dynamic viscosity, $A_{2}=\frac{\rho _{thnf}}{\rho _{f}},\rho _{f}$ represents the density, $A_{3}=\frac{\sigma _{thnf}}{\sigma _{hnf}}$, $A_{4}=\frac{\sigma _{hnf}}{\sigma _{nf}}$, $A_{5}=\frac{\sigma _{nf}}{\sigma _{f}}$, $\sigma _{hnf}$ denotes hybrid nanofluid electrical conductivity, $\sigma _{nf}$ represents nanofluid electrical conductivity, and $\sigma _{f}$ represents base fluid electrical conductivity, $M= \frac{\sigma _{f}{B_{0}}^2}{a_{0}\rho _{f}}$ denotes magnetic parameter, $We^2=\frac{\Gamma ^{2}{\tilde{u}_{w}}^{2}a_0}{1-a\tilde{t}}$ is the same as $\Gamma ^{2}Re_{\tilde{x}}\left( \frac{a_0}{1-a\tilde{t}}\right) ^{2}$ denotes the Weissenberg number, $Re_{\tilde{x}}= \frac{\tilde{x}\tilde{u}_w}{\nu _{f}}$ stands for the Reynolds number, $E_{1}=\frac{E_{0}}{\tilde{u}_w B_{0}}$ represents the electric field parameter, $\delta = \frac{a}{a_{0}}.$ represents the unsteadiness parameter, $A_{6}=\frac{\text {k}_{thnf}}{\text {k}_{hnf}}$, $A_{7}=\frac{\text {k}_{hnf}}{\text {k}_{nf}}$, $A_{8}=\frac{\text {k}_{nf}}{\text {k}_{f}}$, where $\text {k}_{hnf}$ symbolizes the thermal conductivity of the hybrid nanofluid, $A_{9} = \frac{(\rho c_{p})_{thnf}}{(\rho c_{p})_{f}}$, $(c_{p})_{f}$ denotes the specific heat under constant pressure, $A_{10}=\frac{D_{thnf}}{D_{f}}$ denotes mass diffusivity, $Ec=\frac{\tilde{u}_w^{2}}{(c_{p})_{f}\left( \tilde{T}_{w}-\tilde{T}_{\infty }\right) }$the symbol stands for Eckert number, $Rd=\frac{4\tilde{\sigma }{\tilde{T}_{\infty }}^{3}}{\tilde{k}\text {k}_{f}}$ stand for Radiation parameter,$Pr=\frac{{(c_{p})_{f}}\mu _{f}}{\text {k}_{f}}$ stands for the Prandtl number, $Q=\frac{Q_{0}(1-a\tilde{t})}{a_{0}(\rho c_{p})_{f}}$ stands for heat generating parameter, $Sc=\frac{\nu _f}{D_f}$ denotes Schmidt number, $k_{r}=\frac{\text {K}(1-a\tilde{t})}{a_0}$ chemical reaction parameter.

Key Boundaries:

12 $$\begin{aligned} \begin{aligned}&f(0) = S, \quad f'(0) = 1, \quad \theta '(0) = -\frac{Bi \left[ 1 - \theta (0)\right] }{A_6 A_7 A_8}, \quad \phi (0) = 1, \\&f'(\infty ) \rightarrow 0, \quad \theta (\infty ) \rightarrow 0, \quad \phi (\infty ) \rightarrow 0. \end{aligned} \end{aligned}$$

where *S* = $\frac{\tilde{v}_w}{\frac{a_0}{1-a\tilde{t}} \nu _f}$ represents the Suction parameter, $Bi=\frac{h_{f}}{k_{f}} \sqrt{\frac{\nu _{f}(1-a\tilde{t})}{a_{0}}}$, represents the Biot number Tables 1 and 2.

### Physical quantities from an engineering approach

Engineering parameters such as the dimensionless skinfriction coefficient (Cfx), Nusselt numbers (Nux), and Sherwood numbers (Shx) reflect shear stress and heat rate, respectively.(by following Ramzan et al. [54] and [36])

13 $$\begin{aligned} C_{fx}=\frac{\tau _{w}}{\rho _{f}{\tilde{u}_{w}}^{2}}, Nu_{x}=\frac{\tilde{x}q_{w}}{K_{f}(\tilde{T_w}-\tilde{T_{\infty }})}, Sh_{x}=\frac{\tilde{x} q_{m}}{D_{f}(\tilde{C_{w}}-\tilde{C_{\infty }})} \end{aligned}$$

Here $\tau _{w}$, $q_{w}$ and $q_{m}$ are:

14 $$\begin{aligned} \begin{aligned} \tau _{w}&= \left\{ \mu _{thnf} \left( \frac{\partial \tilde{u}}{\partial \tilde{y}} \right) \left[ 1 + \Gamma ^{2} \left( \frac{\partial \tilde{u}}{\partial \tilde{y}} \right) ^{2} \right] ^{\frac{n-1}{2}} \right\} _{y=0}, \\ q_{w}&= -\text {k}_{thnf} + \frac{16 \tilde{\sigma } \tilde{T_{\infty }}^{3}}{3 \tilde{k}} \left( \frac{\partial \tilde{T}}{\partial \tilde{y}} \right) _{y=0}, \\ q_{m}&= -D_{thnf} \left( \frac{\partial \tilde{C}}{\partial \tilde{y}} \right) _{y=0} \end{aligned} \end{aligned}$$

where $\tau _w$ is the wall shear stress, $q_w$ is the surface heat flux, and $q_m$ is the surface mass flux. using (8) and (14), we get

15 $$\begin{aligned} \begin{aligned} Cf_x {Re_{x}}^{1/2}&= A_1 f''(0) \left\{ 1 + We^2 \left( f''(0) \right) ^2 \right\} ^{\frac{n-1}{2}}, \\ Nu_x {Re_{x}}^{-1/2}&= -\left( A_6 A_7 A_8 + \frac{4}{3} R_d \right) \theta '(0), \\ Sh_x {Re_{x}}^{-1/2}&= -A_{10}\phi '(0) \end{aligned} \end{aligned}$$

where $Re_{x}=\frac{\tilde{x}\tilde{u}_{w}}{\nu _{f}}$ is the local Reynolds number, $f(\eta )$ is the dimensionless stream function, $\theta (\eta )$ is the dimensionless temperature, and $\phi (\eta )$ is the dimensionless concentration. It should be noted that the thermal radiation parameter *Rd* contributes directly to the energy balance near the wall, thereby affecting the surface heat flux $q_w$. Since the Nusselt number is defined based on $q_w$, the inclusion of *Rd* is essential to capture radiative heat transfer in high-temperature applications accurately. The parameters are calculated numerically and presented in Section 5 (Tables 3, 4, and 5). The influence of physical parameters on wall shear, heat transfer, and species diffusion is quantified, serving as primary thermophysical indicators of system performance.

### Physical characteristics of ternary hybrid nanofluids

The properties of the ternary hybrid nanofluid are developed step by step: starting from the base fluid, then moving to mono nanofluid (Cu), followed by binary hybrid (Cu + $\hbox {Al}_2\hbox {O}_3$), and finally to ternary hybrid (Cu + $\hbox {Al}_2\hbox {O}_3$ + $\hbox {TiO}_2$). The extended Brinkman [55], Pak-Cho [56], and Maxwell [57] models describe viscosity, density, specific heat, thermal conductivity, and electrical conductivity based on the volume fractions of nanoparticles ($\chi _1$, $\chi _2$, $\chi _3$). This setup ensures that the role of each particle is clearly shown.

#### Dynamic viscosity

$$\begin{aligned} {\mu _{\text {thnf}}} = \frac{1}{(1 - \chi _1)^{2.5}(1 - \chi _2)^{2.5}(1 - \chi _3)^{2.5}} \times \mu _f \end{aligned}$$

#### Density

$$\begin{aligned} \rho _{\text {thnf}} = \left( 1-\chi _{3}\right) \bigg [(1 - \chi _2) \bigg \{(1 - \chi _1) + \frac{\chi _1 \rho _1}{\rho _f}\bigg \}+\frac{\chi _2 \rho _2}{\rho _f}\bigg ] + \frac{\chi _3 \rho _3}{\rho _f} \end{aligned}$$

#### Specific heat

$$\begin{aligned} (\rho C_p)_{\text {thnf}} = (1 - \chi _3) \bigg [(1 - \chi _2) \bigg \{\left( 1-\chi _{1}\right) +\frac{\chi _1 (\rho C_p)_1}{(\rho C_p)_f}\bigg \}+\frac{\chi _2 (\rho C_p)_2}{(\rho C_p)_f}\bigg ] + \frac{\chi _3 (\rho C_p)_3}{(\rho C_p)_f} \end{aligned}$$

#### Thermal conductivity

$$\begin{aligned} k_{\text {thnf}} = \frac{2k_{\text {hnf}} + k_3 + 2\chi _3 (k_3 - k_{\text {hnf}})}{2k_{\text {hnf}} + k_3 - \chi _3 (k_3 - k_{\text {hnf}})} \times k_{\text {hnf}} \end{aligned}$$

where

$$\begin{aligned} k_{\text {hnf}} = \frac{2k_{\text {nf}} + k_2 + 2\chi _2 (k_2 - k_{\text {nf}})}{2k_{\text {nf}} + k_2 - \chi _2 (k_2 - k_{\text {nf}})} \times k_{\text {nf}} \end{aligned}$$

and

$$\begin{aligned} k_{\text {nf}} = \frac{2k_f + k_1 + 2\chi _1 (k_1 - k_f)}{2k_f + k_1 - \chi _1 (k_1 - k_f)} \times k_f \end{aligned}$$

#### Electrical conductivity

$$\begin{aligned} \sigma _{\text {thnf}} = \frac{-2\chi _3 (\sigma _{\text {hnf}} - \sigma _3) + \sigma _3 + 2\sigma _{\text {hnf}} }{\chi _3 (\sigma _{\text {hnf}} - \sigma _3) + 2\sigma _{\text {hnf}} +\sigma _3 }\times \sigma _{\text {hnf}} \end{aligned}$$

where

$$\begin{aligned} \sigma _{\text {hnf}} = \frac{-2\chi _2 (\sigma _{\text {nf}} - \sigma _2) + \sigma _2 + 2\sigma _{\text {nf}}}{\chi _2 (\sigma _{\text {nf}} - \sigma _2) + \sigma _2 + 2\sigma _{\text {nf}}} \times \sigma _{\text {nf}} \end{aligned}$$

and

$$\begin{aligned} \sigma _{\text {nf}} = \frac{-2\chi _1 (\sigma _f - \sigma _1) + \sigma _1 + 2\sigma _f}{\chi _1 (\sigma _f - \sigma _1) + \sigma _1 + 2\sigma _f} \times \sigma _f \end{aligned}$$

#### Mass diffusivity

$$\begin{aligned} D_{thnf} = D_{f} (1-\chi _{1}) (1-\chi _{2}) (1-\chi _{3}) \end{aligned}$$

## Analysis of entropy generation

The irreversible thermal energy produced by some irreversible processes that take place during fluid movement is known as entropy. According to Ref. [36, 54] the volumetric entropy production rate in the Carreau ternary hybrid nanofluid as a result of heat radiation is described as follows:

16 $$\begin{aligned} \begin{aligned} H_{\text {Gen}}'''&= \frac{\mu _{\text {thnf}}}{\tilde{T}_\infty } \left( \frac{\partial \tilde{u}}{\partial \tilde{y}} \right) ^2 \left\{ 1 + \Gamma ^2 \left( \frac{\partial \tilde{u}}{\partial \tilde{y}} \right) ^2 \right\} ^{\frac{n-1}{2}} + \frac{1}{\tilde{T_\infty }^2} \left( k_{\text {thnf}} + \frac{16}{3} \tilde{\sigma } \tilde{T}^{3} \tilde{k}^{-1} \right) \left( \frac{\partial \tilde{T}}{\partial \tilde{y}} \right) ^2 \\&\quad + \frac{\sigma _{\text {thnf}}}{\tilde{T}_\infty } \left( \tilde{u} B -E\right) ^{2} \end{aligned} \end{aligned}$$

The characteristic entropy formation rate is:

17 $$\begin{aligned} H_0''' = k_f \left( \frac{\tilde{T}_{w} - \tilde{T}_\infty }{\tilde{T}_\infty } \right) ^2 \left( \frac{\eta }{\tilde{y}} \right) ^2 \end{aligned}$$

Using (8), (16), (17) the dimensionless entropy generation is expressed as:

18 $$\begin{aligned} \begin{aligned} \text {Ng}&= \frac{H_{\text {Gen}}'''}{H_0'''} = A_{1} \frac{Br}{\beta _{1}} f''^{2} \left\{ 1 + We^{2} f''^{2} \right\} ^{\frac{n-1}{2}} + \left[ A_{6} A_{7} A_{8} + \frac{4}{3} R_d \right] \theta '^{2} \\&\quad + A_{3} A_{4} A_{5} M \frac{Br}{\beta _1} \left( f' - E_1 \right) ^{2} \end{aligned} \end{aligned}$$

where $\beta _{1} = \frac{\tilde{T}_{w}-\tilde{T}_{\infty }}{\tilde{T}_{\infty }}$, denotes the dimensionless temperature ratio parameter, $\text {Br} = \frac{\mu _{f} {\tilde{u_w}}^2}{k_f \left( \tilde{T}_{w}-\tilde{T}_{\infty }\right) }$ describes the Brinkman number.

The Bejan number is the ratio of the irreversibility of heat transport to the total entropy production rate. One example of a non-dimensional Bejan number is:

19 $$\begin{aligned} \text {Be} = \frac{\left( A_{6} A_{7} A_{8} + \frac{4}{3} Rd \right) \theta ^{\prime 2}}{\text {Ng}}. \end{aligned}$$

## Numerical procedure

### Solution methodology

Using MATLAB’s fifth-order Runge–Kutta-Fehlberg approach, the non linear and coupled ODE system equations:(9), (10), (11) is numerically solved. Boundary layer conditions are fulfilled by reducing higher-order derivatives to a first-order system with a step size of 0.01, a far-field boundary condition $\eta _{\infty }=10$, and a convergence tolerance of $10^{-6}$. To confirm the robustness of the numerical solution, a mesh refinement study was carried out. The number of collocation points was varied from 200 to 1000, and the solutions for velocity, temperature, and concentration profiles were compared. The results were found to be mesh-independent, with deviations less than $10^{-4}$ between successive refinements. This validates that the solver achieved stable convergence and that the reported solutions are numerically reliable. In addition, the obtained solutions were cross-verified with previously published works [58–60], showing excellent agreement and further confirming the accuracy of the BVP5c implementation. The BVP5c technique solves the system obtained from PDEs using proper transformations and boundary conditions equation:(12) by reformulating it into first-order equations.

20 $$\begin{aligned} \begin{aligned}&S_1 = f, \quad S_2 = f', \quad S_3 = f'', \quad {S_3}' = f''', \\&S_4 = \theta , \quad S_5 = \theta ', \quad {S_5}' = \theta '', \\&S_6 = \phi , \quad S_7 = \phi ', \quad {S_7}' = \phi ''. \end{aligned} \end{aligned}$$

The first order-based system was obtained:

21 $$\begin{aligned} \begin{aligned}&{S_1}' = S_2, \\&{S_2}' = S_3, \\&{S_3}' = \frac{\frac{A_{3}A_{4}A_{5}}{A_{2}}M\left[ E_{1} - S_{2}\right] + \delta \left[ S_{3}\frac{\eta }{2} + S_{2}\right] + {S_{2}}^{2} - S_{1}S_{3}}{\frac{A_{1}}{A_{2}} \left[ \left\{ 1 + We^{2} S_{3}^{2} \right\} ^{\frac{n-1}{2}} + \left( n-1\right) We^{2} S_{3}^{2} \left\{ 1 + We^{2} S_{3}^{2} \right\} ^{\frac{n-3}{2}}\right] }, \\&{S_{4}}' = S_5\\&{S_{5}}'=\frac{A_{9}Pr\delta \left[ S_{5}\frac{\eta }{2}+S_{4}\right] +A_{9}PrS_{2}S_{4}-A_{9}PrS_{1}S_{5}-A_{3}A_{4}A_{5}MEcPr\left[ S_{2}-E1\right] ^{2}-QPrS_{4}}{\left[ A_{6}A_{7}A_{8}+\frac{4}{3}Rd\right] }\\&{S_{6}}'=S_7,\\&{S_{7}}'=\frac{\delta Sc \left[ S_{7} \left( \frac{\eta }{2} \right) + S_{6}\right] +Sc S_{2}S_{6}-Sc S_{1}S_{7}- K_{r} Sc S_{6}}{A_{10}} \end{aligned} \end{aligned}$$

and the accompanying boundary conditions:

22 $$\begin{aligned} \begin{aligned}&S_{1}(0) = S, \quad S_{2}(0) = 1, \quad S_{5}(0) = -\frac{Bi}{A_{6}A_{7}A_{8}}\left[ 1 - S_{4}(0)\right] , \quad S_{6}(0) = 1, \\&S_{2}(\infty ) = 0, \quad S_{4}(\infty ) = 0, \quad S_{6}(\infty ) = 0. \end{aligned} \end{aligned}$$

### Validation of the solution

To calculate the values of $-f''(0)$, we altered the unsteadiness parameter $\delta$, which is exclusive to this investigation. We set $We = M = E_{1} = 0$ and $n = 1$ for these computations. We were able to verify and corroborate the study’s mathematical results using this method. The purpose of these computations is to validate and verify the derived mathematical findings. To assess the precision and coherence of the mathematical answers, the test simulates this decision-making process. The assessed values were connected to the research conducted by Mukhopadhyay et al. [58], Chamakha et al. [59], and Sharidan et al. [60]. Table 2 provides a significant graphical depiction of this association.

**Table 2:** Verification $-f''(0)$ for varying $\delta$ values

$\delta$	Sharidan et al. [60]	Chamakha et al. [59]	Mukhopadhyay et al. [58]	Present result
0.8	1.261042	1.261512	1.261479	1.2610737
1.2	1.377722	1.378052	1.377850	1.3774207

The data analysis and examples provided in the tables show a significant degree of consistency. They demonstrated that the research they compiled was reliable and precise. Furthermore, it was certified that the computer code had been rectified, that information regarding this mathematical method was created, and that it had been chosen.

## Results and discussion

This section of the research examines the impact of active factors on energy, velocity, concentration, skin friction coefficient, heat transfer rate, mass transfer rate, entropy production, and Bejan number, accompanied by pertinent graphs. The ternary hybrid nanofluid ($\hbox {TiO}_2$ + Cu + $\hbox {Al}_2\hbox {O}_3$/CMC–$\hbox {H}_2$O) shows better heat and mass transfer rates than the binary (Cu + $\hbox {Al}_2\hbox {O}_3$/CMC–$\hbox {H}_2$O) and mono (Cu/CMC–$\hbox {H}_2$O) systems. This improvement comes from the combined effects of Cu’s high thermal conductivity, $\hbox {Al}_2\hbox {O}_3$’s structural stability, and $\hbox {TiO}_2$’s ability to enhance radiation. Therefore, the ternary model offers the best thermal performance while maintaining controlled irreversibility and better rheological stability. The performance parameter settings are established within the used range to optimise the outcomes and align with previously published research.Specifically, the Eckert number (*Ec*) was varied between 0.1 and 0.5, the Biot number (*Bi*) between 0.1 and 2.0, the heat generation/absorption parameter (*Q*) between $-0.5$ and 0.5, the radiation parameter (*Rd*) between 0.5 and 2.0, and the Weissenberg number (*We*) between 0.1 and 2.0, the Brinkman number (*Br*) in the range 0.1–1.0, and the Schmidt number (*Sc*) between 0.5 and 3.0, unsteadiness parameter ($\delta$) was chosen between 0.1 and 0.5, the magnetic parameter (*M*) between 0.0 and 2.0,the electric field paramter $E_1$ and temperature ratio parameter $\beta _1$ were considered in the ranges $-0.5 \le E_1 \le 0.5$ and $0.1 \le \beta _1 \le 0.5$. The nanoparticle volume fractions ($\chi _1, \chi _2, \chi _3$) were taken in the dilute suspension range 0.01–0.04, the chemical reaction parameter ($K_r$) between 0.0 and 0.5, and the Suction parameter (*S*) between 0.0 and 1.0. Unless specified otherwise, the following fixed values were used for the simulations: $Ec=0.3$, $Bi=0.5$, $Q=-0.1$, $Pr=6.2$, $Rd=0.5$, $n=1.5$, $Br=0.5$, $Sc=2.0$, $\delta =0.2$, $M=0.2$, $E_1=-0.1$, $\beta _1=0.2$, $We=0.2$, $\chi _1=\chi _2=\chi _3=0.02$, $K_r=0.5$, and $S=0.5$.

### Velocity profile

**Fig. 2:**
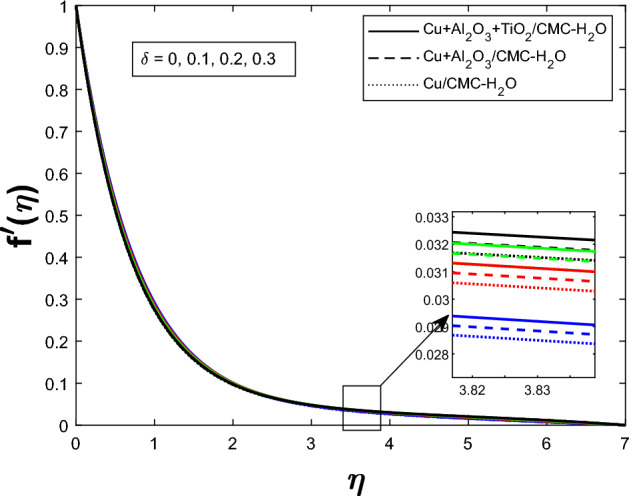
Velocity profile $f'(\eta )$ for Unsteadiness parameter $\delta$

**Fig. 3:**
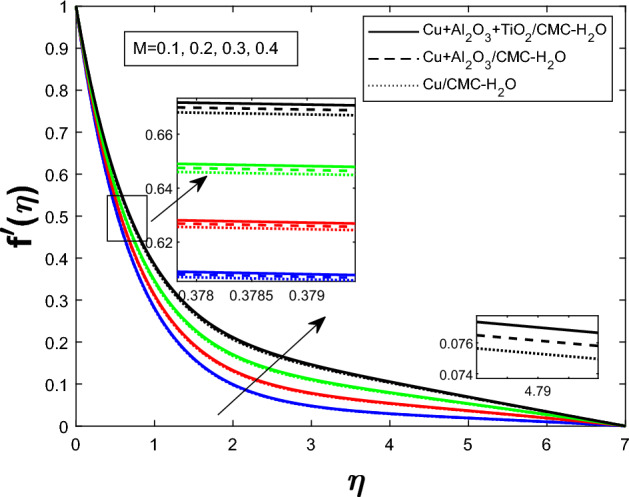
Velocity profile $f'(\eta )$ for Magnetic parameter *M*

**Fig. 4:**
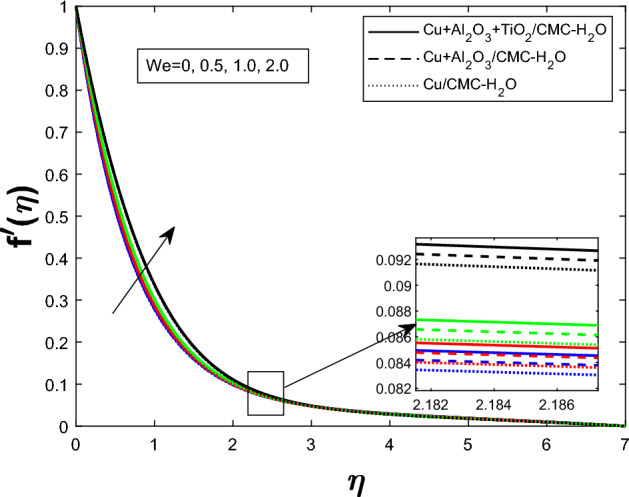
Velocity profile $f'(\eta )$ for Weissenberg numbers *We*

**Fig. 5:**
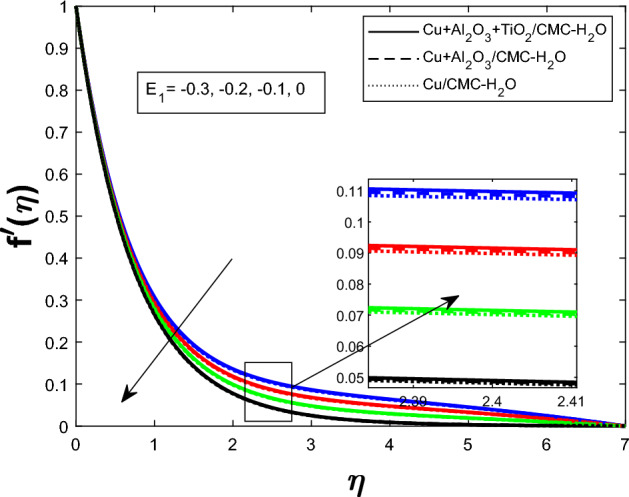
Velocity profile $f'(\eta )$ for Electric field parameter $E_1$

**Fig. 6:**
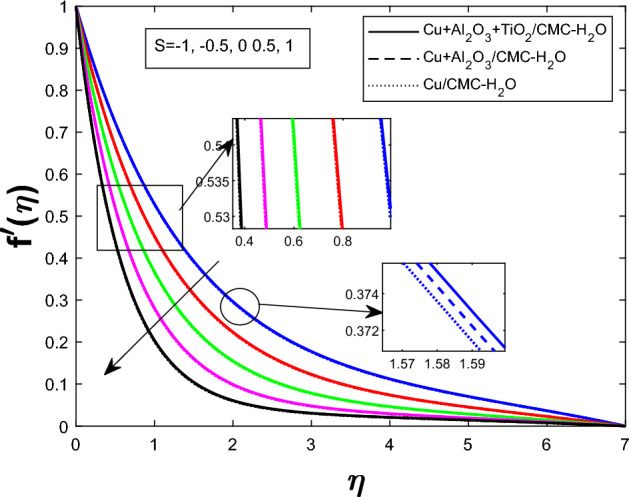
Velocity profile $f'(\eta )$ for Suction parameter *S*

The Fig. 2 illustrates how the unsteadiness parameter $$\delta $$ affects fluid velocity Fluid velocity declines with increasing $$\delta $$, resulting in a narrower velocity boundary layer near the wall and a flatter velocity profile farther away. In Fig. 3, the magnetic parameter *M* impacts velocity distribution. The velocity profile improves with larger *M*, demonstrating that the Lorentz force enhances fluid motion under current circumstances. Figure 4 shows how the Weissenberg number *We* affects the velocity profile $$f'(\eta )$$. The velocity profile rises as *We* grows, reflecting longer particle relaxation time and increased fluid elasticity, which boosts velocities despite viscous resistance. In Fig. 5, $$f'(\eta )$$ is shown for several values of the electric field parameter $$E_1$$ Velocity falls as $$E_1$$ rises, suggesting a stronger electric field inhibits fluid movement. Figure 6 shows how the suction parameter *S* affects velocity distribution. Higher suction lowers boundary layer thickness, accelerating fluid at the surface and increasing velocity. Figures 2, 3, 4, 5, and 6 demonstrate that fluid velocity is more important in the $$Cu+Al_{2}O_{3} + TiO_{2}/CMC $$-water ternary hybrid nanofluid than in the $$Cu+Al_{2}O_{3}/CMC $$-water hybrid nanofluid.

### Temperature profile

**Fig. 7:**
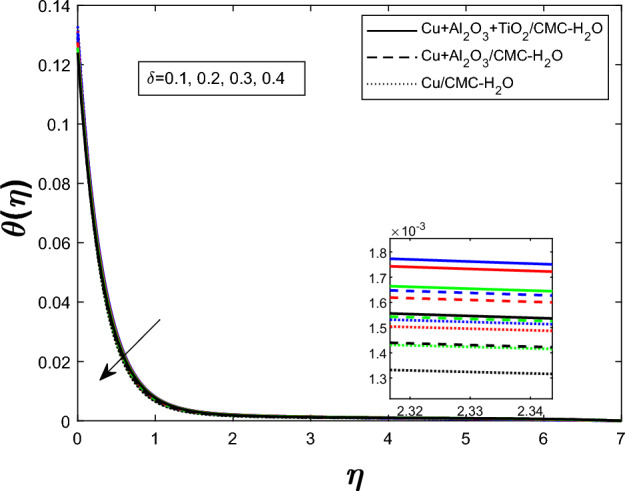
Temperature profile $\theta (\eta )$ for unsteadiness parameters $\delta$

**Fig. 8:**
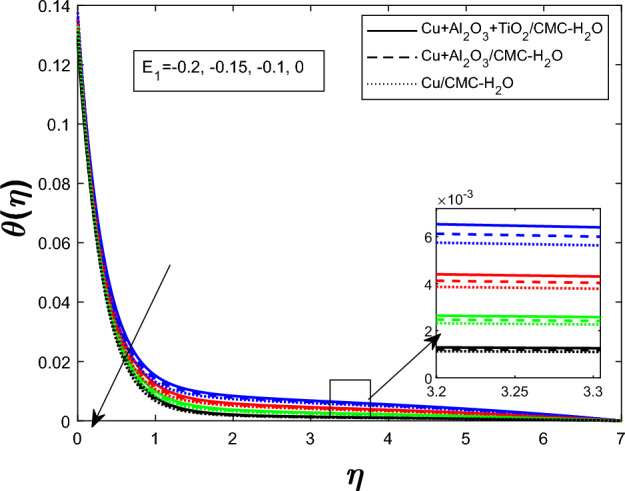
Temperature profile $\theta (\eta )$ for electric field parameters $E_1$

**Fig. 9:**
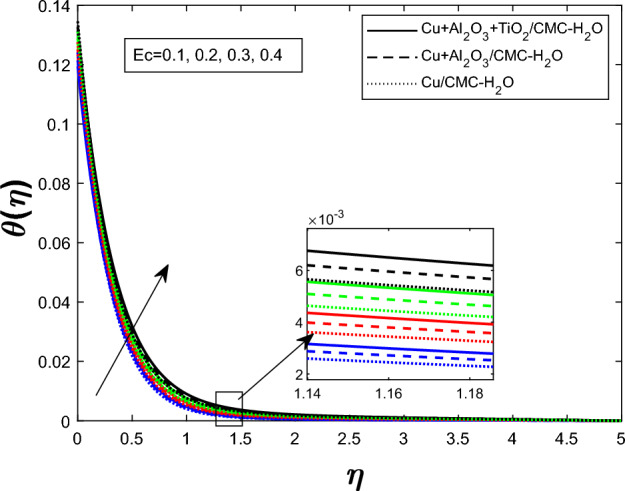
Temperature profile $\theta (\eta )$ for Eckert number *Ec*

**Fig. 10:**
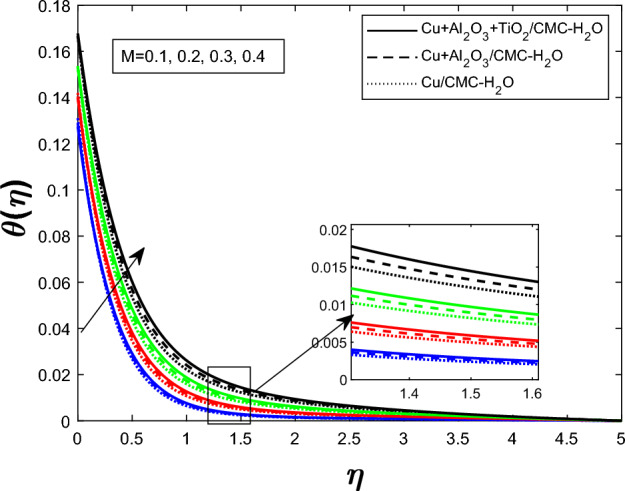
Temperature profile $\theta (\eta )$ for magnetic parameters *M*

**Fig. 11:**
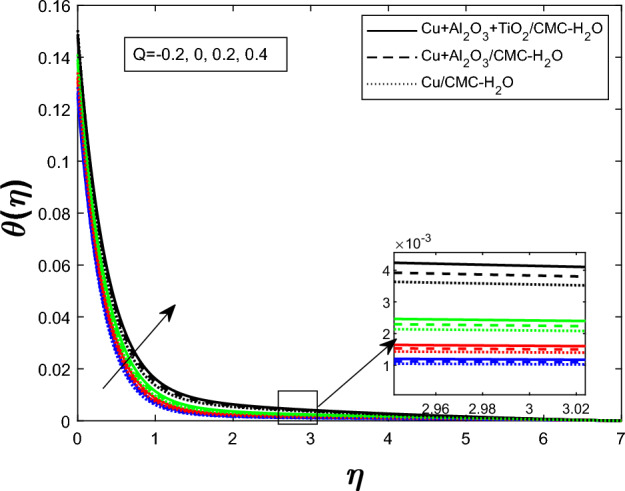
Temperature profile $\theta (\eta )$ for heat source/sink parameters *Q*

**Fig. 12:**
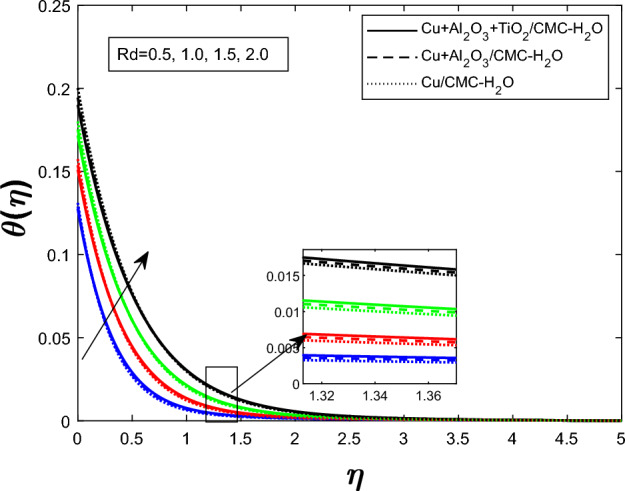
Temperature profile $\theta (\eta )$ for radiation parameters *Rd*

Figure 7 shows how the unsteadiness parameter $$\delta $$ affects the temperature distribution $$\theta (\eta )$$. As $$\delta $$ rises, fluid temperature falls. Increased unsteadiness increases nanoparticle mobility and heat dissipation. Figure 8 for the impact of the electric field parameter $$E_1$$ on $$\theta (\eta )$$ The electric field suppresses thermal energy, limiting resistive heating under the examined circumstances, lowering the temperature profile as $$E_1$$ increases. Figure  9 illustrates the impact of Eckert number *Ec* on temperature. Viscous dissipation creates internal heat, improving the temperature profile as *Ec* increases. Figure 10 shows how the magnetic parameter *M* affects $$\theta (\eta )$$. The Lorentz force opposing fluid motion generates thermal energy via resistive effects, raising the temperature as *M* increases. Figure  11 illustrates how increasing heat source parameter *Q* results in higher temperatures. The thermal boundary layer thickens as a result of more energy being injected into the system by higher internal heat production. Finally, Fig. 12 shows the significance of the thermal radiation parameter *Rd*. More thermal radiation increases energy transfer to fluid molecules and thermal boundary layer thickness, raising fluid temperature. Figures 7, 8, 9, 10, 11, and 12 show that the $$Cu+Al_{2}O_{3}+TiO_{2}/CMC $$-water ternary hybrid nanofluid has a higher fluid temperature than the $$Cu+Al_{2}O_{3}/CMC $$-water hybrid nanofluid.

### Concentration profile

**Fig. 13:**
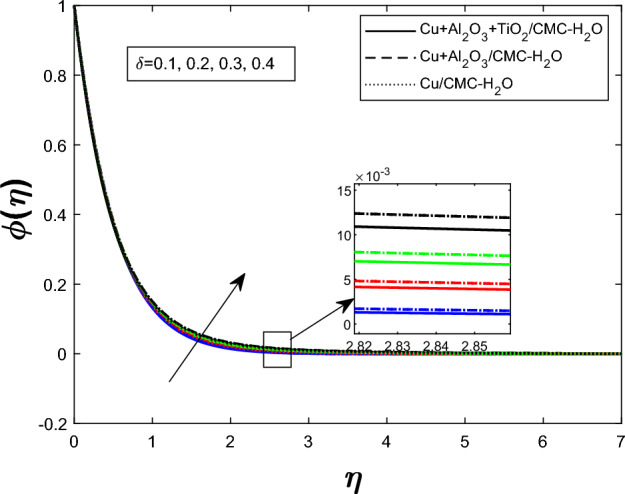
Concentration profile $\phi (\eta )$ for unsteadiness parameter $\delta$

**Fig. 14:**
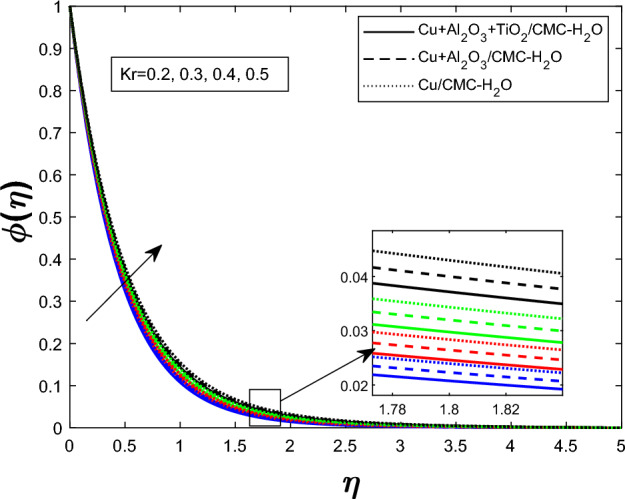
Concentration profile $\phi (\eta )$ for schmidt number *Sc*

**Fig. 15:**
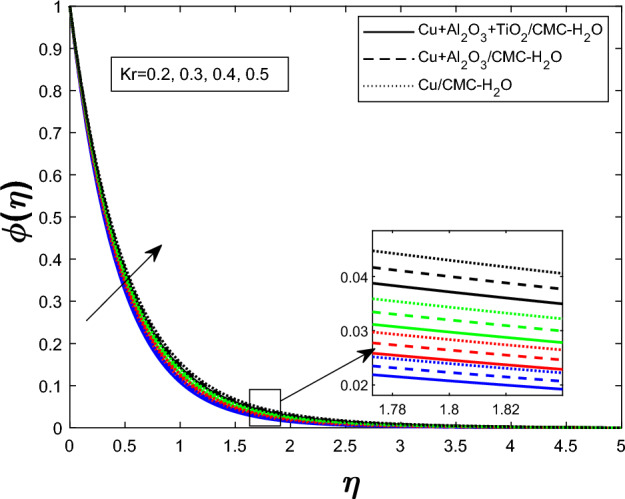
Concentration profile $\phi (\eta )$ for chemical reaction Parameter $K_r$

The concentration profile $\phi (\eta )$ for varying values of the unsteadiness parameter $\delta$ is shown in Fig. 13. When $\delta$ rises, so does $\phi (\eta )$. The impact of the Schmidt number $Sc$ on $\phi (\eta )$ is seen in Fig. 14. Reduced molecular diffusivity slows solute transport and promotes greater local concentrations, hence a rise in $Sc$ improves the concentration profile. Figure 15 demonstrates that when stronger chemical reactions produce or retain more species within the fluid, raising the chemical reaction parameter $K_r$ likewise rises $\phi (\eta )$. The ternary hybrid nanofluid ($\textrm{Cu} + \textrm{Al}_2\textrm{O}_3 + \textrm{TiO}_2/\textrm{CMC}$-water) maintains a higher concentration profile than the binary hybrid nanofluid ($\textrm{Cu} + \textrm{Al}_2\textrm{O}_3/\textrm{CMC}$-water), as shown collectively in Figs. 13, 14, and 15.

### Entropy generation and Bejan number

**Fig. 16:**
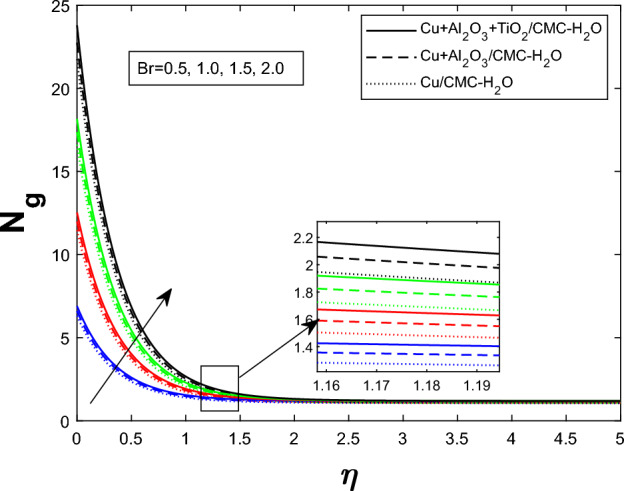
Producing entropy in relation to *Br*

**Fig. 17:**
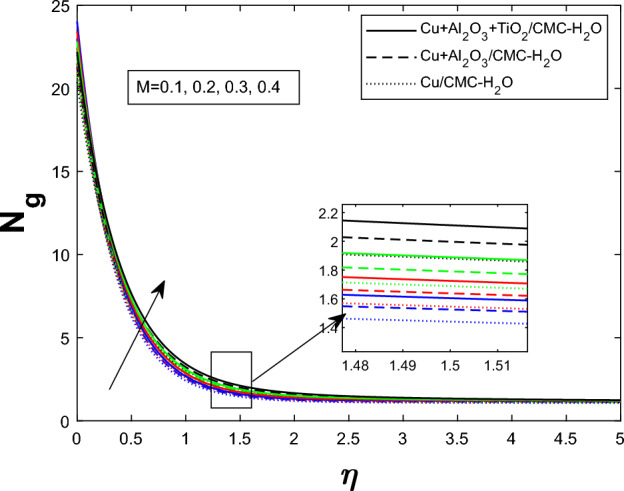
Producing entropy in relation to *M*

**Fig. 18:**
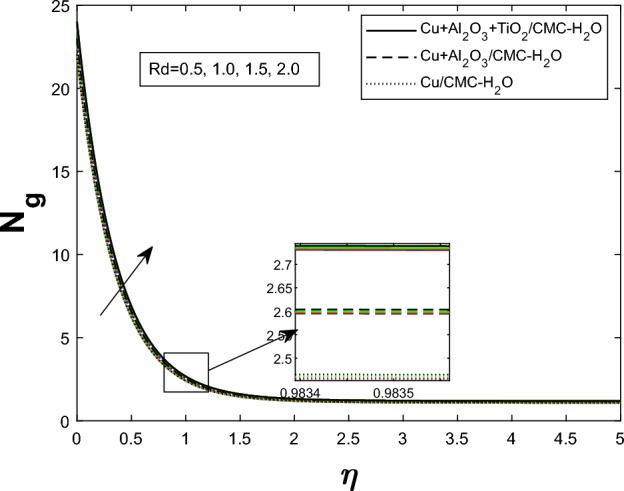
Producing entropy in relation to *Rd*

**Fig. 19:**
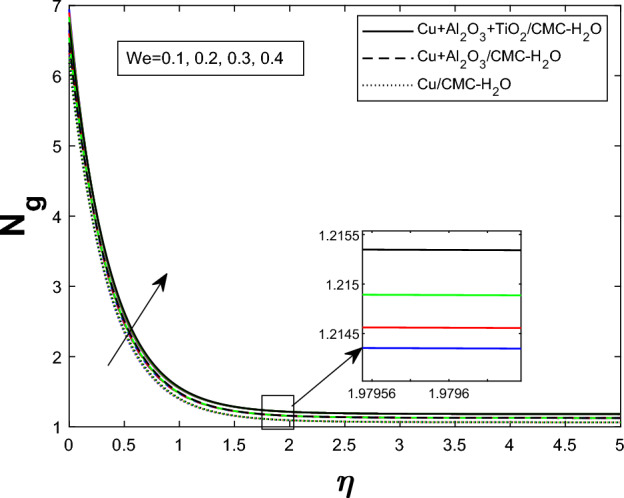
Producing entropy in relation to *We*

Entropy generation $N_g$ varies for many parameters, including Brinkman number $Br$, magnetic parameter $M$, radiation parameter $Rd$, and Weissenberg number $We$, as shown in Figs. 16, 17, 18, and 19. As seen in Fig. 16, raising $Br$ increases $N_g$ by increasing viscous dissipation, which turns mechanical energy into heat and increases system irreversibility. As $M$ increases, the Lorentz force, Ohmic heating, and viscous resistance increase, leading to increased entropy generation (see Fig. 17). Higher thermal radiation (Rd) increases temperature gradients, enhancing thermal irreversibility and raising $N_g$ (see Fig. 18). Figure 19 shows that $N_g$ increases with $We$ due to increased internal friction and energy dissipation due to greater elastic effects. Entropy generation is maximum in the tri hybrid nanofluid $Cu+Al_2O_3+TiO_2/CMC-water$ compared to binary and mono nanofluids. Increased entropy production increases irreversibility and fluid motion via thermal and momentum transfer processes.

**Fig. 20:**
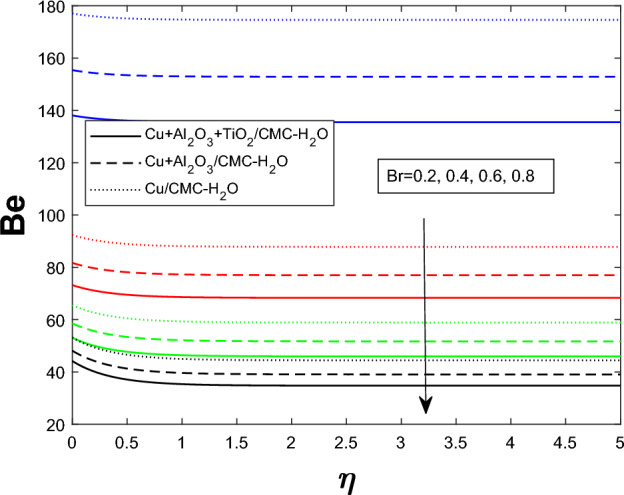
Bejan number against Br

**Fig. 21:**
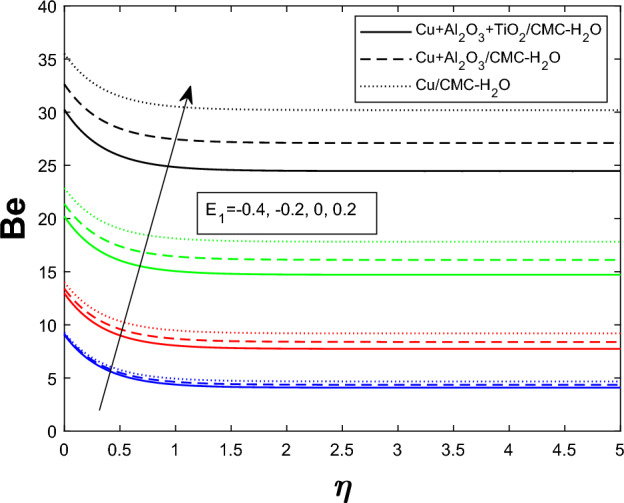
Bejan number against $E_1$

**Fig. 22:**
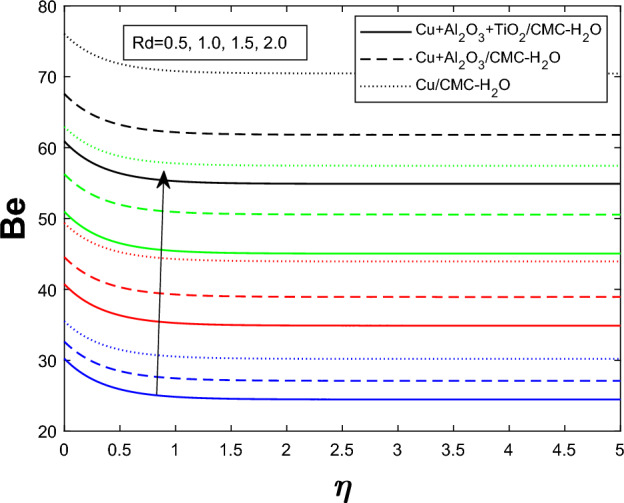
Bejan number against Rd

Figures 20, 21, and 22 show how the ternary hybrid nanofluid’s Bejan number $Be$ varies depending on $Br$, $E_1$, and $Rd$, respectively. The percentage contribution from heat transfer is reduced in Fig. 20 as $Be$ drops as the Brinkman number $Br$ increases owing to more viscous dissipation, which increases entropy formation from fluid friction. Figure 21 demonstrates that ( Be ) rises when the electric field parameter $E_1$ increases. This is because higher electric fields reduce frictional entropy and enhance thermal irreversibility by dampening fluid motion. Since more radiative heat transfer amplifies thermal entropy development in comparison to viscous effects, raising the thermal radiation parameter $Rd$ in Fig. 22 improves $Be$. Overall, these patterns show that larger $Br$ moves the balance towards mechanical irreversibility, while thermal irreversibility becomes more important when electric and radiative impacts are stronger

### Streamlines

**Fig. 23:**
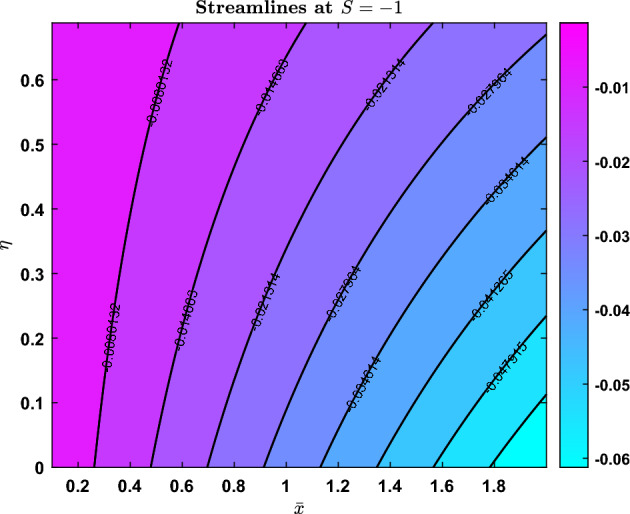
Streamline pattern when S=-1

**Fig. 24:**
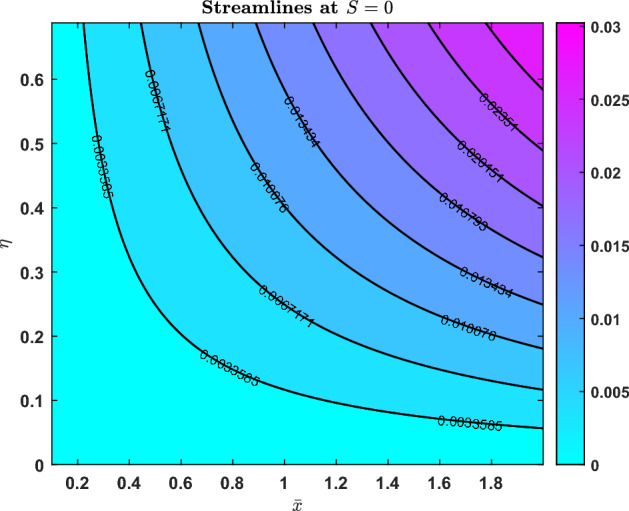
Streamline pattern when S=0

**Fig. 25:**
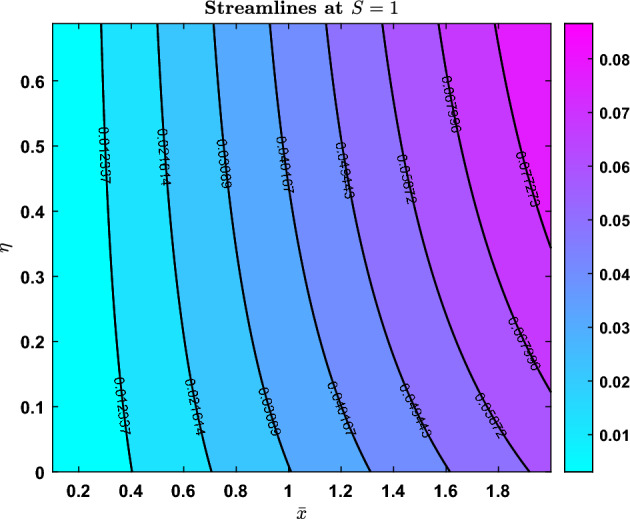
Streamline pattern when S=1

The streamline contours for three distinct values of the parameter $S = -1$, $S = 0$, and $S = 1$ are shown in Figs. 23, 24, and 25. The normalised variable *barx* is represented by the horizontal axis, while *eta* is represented by the vertical axis. The contour lines and colour scale match the stream function’s value.

When $S = -1$, the flow field trends from left (higher) to right (lower), streamlines curve to the right, and the values are negative. A transitional flow state is shown by the stream function changing sign at $S = 0$, with streamlined contours merging between the two regimes. In contrast to the previous situations, $S = 1$ highlights greater flow since the streamlines are angled to the left, all values are positive, and the colour changes.

The varying contours and colour gradients in these patterns imply that the parameter *S* regulates the flow’s strength as well as its direction.

### Engineering parameters

**Table 3:** Variation of skin-friction coefficient $C_f \cdot \sqrt{Re_x}$ with different parameters

*M*	*n*	*We*	*S*	$\delta$	$E_1$	$\chi _1$	$\chi _2$	$\chi _3$	*C*_*f*_ $\sqrt{Re_x}$
0.2	1.5	0.2	0.5	0.2	− 0.1	0.02	0.02	0.02	− 1.4707
0.3	1.5	0.2	0.5	0.2	− 0.1	0.02	0.02	0.02	− 1.3681
0.4	1.5	0.2	0.5	0.2	− 0.1	0.02	0.02	0.02	− 1.2586
0.1	0.5	0.2	0.5	0.2	− 0.1	0.02	0.02	0.02	− 1.5563
0.1	2.5	0.2	0.5	0.2	− 0.1	0.02	0.02	0.02	− 1.5745
0.1	3.5	0.2	0.5	0.2	− 0.1	0.02	0.02	0.02	- 1.5819
0.1	1.5	0.5	0.5	0.2	− 0.1	0.02	0.02	0.02	− 1.5859
0.1	1.5	1.0	0.5	0.2	− 0.1	0.02	0.02	0.02	− 1.6336
0.1	1.5	1.5	0.5	0.2	− 0.1	0.02	0.02	0.02	− 1.6832
0.1	1.5	0.2	− 1.0	0.2	− 0.1	0.02	0.02	0.02	− 0.7697
0.1	1.5	0.2	0.0	0.2	− 0.1	0.02	0.02	0.02	− 1.2259
0.1	1.5	0.2	1.0	0.2	− 0.1	0.02	0.02	0.02	− 1.9744
0.1	1.5	0.2	0.5	0.0	− 0.1	0.02	0.02	0.02	− 1.4590
0.1	1.5	0.2	0.5	0.1	− 0.1	0.02	0.02	0.02	− 1.5174
0.1	1.5	0.2	0.5	0.2	− 0.1	0.02	0.02	0.02	− 1.5658
0.1	1.5	0.2	0.5	0.2	− 0.5	0.02	0.02	0.02	− 1.4710
0.1	1.5	0.2	0.5	0.2	− 0.3	0.02	0.02	0.02	− 1.5154
0.1	1.5	0.2	0.5	0.2	− 0.1	0.02	0.02	0.02	− 1.5396
0.1	1.5	0.2	0.5	0.2	− 0.1	0.01	0.02	0.02	− 1.4915
0.1	1.5	0.2	0.5	0.2	− 0.1	0.02	0.02	0.02	− 1.5658
0.1	1.5	0.2	0.5	0.2	− 0.1	0.03	0.02	0.02	− 1.6402
0.1	1.5	0.2	0.5	0.2	− 0.1	0.02	0.01	0.02	− 1.5317
0.1	1.5	0.2	0.5	0.2	− 0.1	0.02	0.02	0.02	− 1.5658
0.1	1.5	0.2	0.5	0.2	− 0.1	0.02	0.02	0.03	− 1.6004
0.1	1.5	0.2	0.5	0.2	− 0.1	0.02	0.02	0.01	− 1.5294
0.1	1.5	0.2	0.5	0.2	− 0.1	0.02	0.02	0.02	− 1.5658
0.1	1.5	0.2	0.5	0.2	− 0.1	0.02	0.02	0.03	− 1.6026

**Table 4:** Variation of Nusselt number ($Nu_xRe_x^{-1/2}$) with respect to various parameters

*M*	$\delta$	$E_1$	*Rd*	*Pr*	*Ec*	*Q*	*Bi*	$\chi _1$	$\chi _2$	$\chi _3$	Nu_x_Re_x_^-1/2^
0.2	0.2	$-$0.1	0.5	6.2	0.3	$-$0.1	0.5	0.02	0.02	0.02	0.6727
0.3	0.2	$-$0.1	0.5	6.2	0.3	$-$0.1	0.5	0.02	0.02	0.02	0.6609
0.4	0.2	$-$0.1	0.5	6.2	0.3	$-$0.1	0.5	0.02	0.02	0.02	0.6476
0.1	0.0	$-$0.1	0.5	6.2	0.3	$-$0.1	0.5	0.02	0.02	0.02	0.6874
0.1	0.1	$-$0.1	0.5	6.2	0.3	$-$0.1	0.5	0.02	0.02	0.02	0.6822
0.1	0.2	$-$0.1	0.5	6.2	0.3	$-$0.1	0.5	0.02	0.02	0.02	0.6832
0.1	0.2	$-$0.5	0.5	6.2	0.3	$-$0.1	0.5	0.02	0.02	0.02	0.6630
0.1	0.2	$-$0.3	0.5	6.2	0.3	$-$0.1	0.5	0.02	0.02	0.02	0.6746
0.1	0.2	$-$0.2	0.5	6.2	0.3	$-$0.1	0.5	0.02	0.02	0.02	0.6793
0.1	0.2	$-$0.1	1.0	6.2	0.3	$-$0.1	0.5	0.02	0.02	0.02	0.9050
0.1	0.2	$-$0.1	1.5	6.2	0.3	$-$0.1	0.5	0.02	0.02	0.02	1.1175
0.1	0.2	$-$0.1	2.0	6.2	0.3	$-$0.1	0.5	0.02	0.02	0.02	1.3221
0.1	0.2	$-$0.1	0.5	5.5	0.3	$-$0.1	0.5	0.02	0.02	0.02	0.6763
0.1	0.2	$-$0.1	0.5	6.0	0.3	$-$0.1	0.5	0.02	0.02	0.02	0.6813
0.1	0.2	$-$0.1	0.5	6.5	0.3	$-$0.1	0.5	0.02	0.02	0.02	0.6858
0.1	0.2	$-$0.1	0.5	6.2	0.2	$-$0.1	0.5	0.02	0.02	0.02	0.6864
0.1	0.2	$-$0.1	0.5	6.2	0.4	$-$0.1	0.5	0.02	0.02	0.02	0.6799
0.1	0.2	$-$0.1	0.5	6.2	0.5	$-$0.1	0.5	0.02	0.02	0.02	0.6767
0.1	0.2	$-$0.1	0.5	6.2	0.3	$-$0.1	0.5	0.02	0.02	0.02	0.6832
0.1	0.2	$-$0.1	0.5	6.2	0.3	0.0	0.5	0.02	0.02	0.02	0.6809
0.1	0.2	$-$0.1	0.5	6.2	0.3	0.1	0.5	0.02	0.02	0.02	0.6788
0.1	0.2	$-$0.1	0.5	6.2	0.3	$-$0.1	0.2	0.02	0.02	0.02	0.2936
0.1	0.2	$-$0.1	0.5	6.2	0.3	$-$0.1	0.4	0.02	0.02	0.02	0.5594
0.1	0.2	$-$0.1	0.5	6.2	0.3	$-$0.1	0.7	0.02	0.02	0.02	0.9143
0.1	0.2	$-$0.1	0.5	6.2	0.3	$-$0.1	0.5	0.01	0.02	0.02	0.6896
0.1	0.2	$-$0.1	0.5	6.2	0.3	$-$0.1	0.5	0.02	0.02	0.02	0.6832
0.1	0.2	$-$0.1	0.5	6.2	0.3	$-$0.1	0.5	0.03	0.02	0.02	0.6770
0.1	0.2	$-$0.1	0.5	6.2	0.3	$-$0.1	0.5	0.02	0.01	0.02	0.6894
0.1	0.2	$-$0.1	0.5	6.2	0.3	$-$0.1	0.5	0.02	0.02	0.02	0.6832
0.1	0.2	$-$0.1	0.5	6.2	0.3	$-$0.1	0.5	0.02	0.03	0.02	0.6772
0.1	0.2	$-$0.1	0.5	6.2	0.3	$-$0.1	0.5	0.02	0.02	0.01	0.6885
0.1	0.2	$-$0.1	0.5	6.2	0.3	$-$0.1	0.5	0.02	0.02	0.02	0.6832
0.1	0.2	$-$0.1	0.5	6.2	0.3	$-$0.1	0.5	0.02	0.02	0.03	0.6780

**Table 5:** Variation of Sherwood number ($Sh_xRe_x^{-1/2}$) with respect to various parameters

$\delta$	$K_r$	*Sc*	$\chi _1$	$\chi _2$	$\chi _3$	Sh_x_Re_x_^-1/2^
0.0	0.5	2.0	0.02	0.02	0.02	4.2896
0.1	0.5	2.0	0.02	0.02	0.02	2.6007
0.2	0.5	2.0	0.02	0.02	0.02	2.2964
0.2	0.2	2.0	0.02	0.02	0.02	2.0577
0.2	0.3	2.0	0.02	0.02	0.02	2.0116
0.2	0.4	2.0	0.02	0.02	0.02	2.0228
0.2	0.5	1.5	0.02	0.02	0.02	1.7980
0.2	0.5	2.5	0.02	0.02	0.02	2.7550
0.2	0.5	3.5	0.02	0.02	0.02	3.5979
0.2	0.5	2.0	0.01	0.02	0.02	2.2607
0.2	0.5	2.0	0.02	0.02	0.02	2.2964
0.2	0.5	2.0	0.03	0.02	0.02	2.3299
0.2	0.5	2.0	0.02	0.01	0.02	2.3067
0.2	0.5	2.0	0.02	0.02	0.02	2.2964
0.2	0.5	2.0	0.02	0.03	0.02	2.2826
0.2	0.5	2.0	0.02	0.02	0.01	2.3033
0.2	0.5	2.0	0.02	0.02	0.02	2.2964
0.2	0.5	2.0	0.02	0.02	0.03	2.2872

The effects of different parameters on $C_f\sqrt{Re_x}$, $Nu_x Re_x^{-1/2}$, and $Sh_x Re_x^{-1/2}$ are summarised in Tables 3, 4, and 5. While *We*, *n*, *S*, *Rd*, *Pr*, and *Bi* increase wall shear and heat transfer, increased *M* decreases them. The volume fractions of nanoparticles $\chi _1$, $\chi _2$, and $\chi _3$ show non-linear effects on mass and heat transmission. Transport properties are improved by stretching, stratification, and increased *Sc*. The optimisation of nanofluids in thermal and concentration boundary layer processes is supported by these findings.

**Fig. 26:**
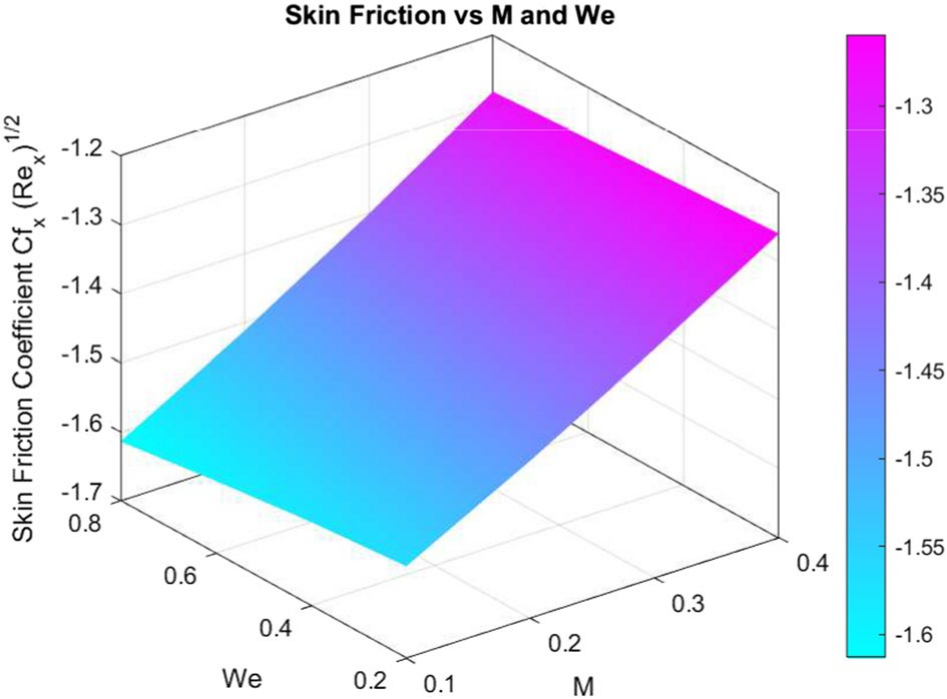
Skin friction coefficient against *M* and *We*

**Fig. 27:**
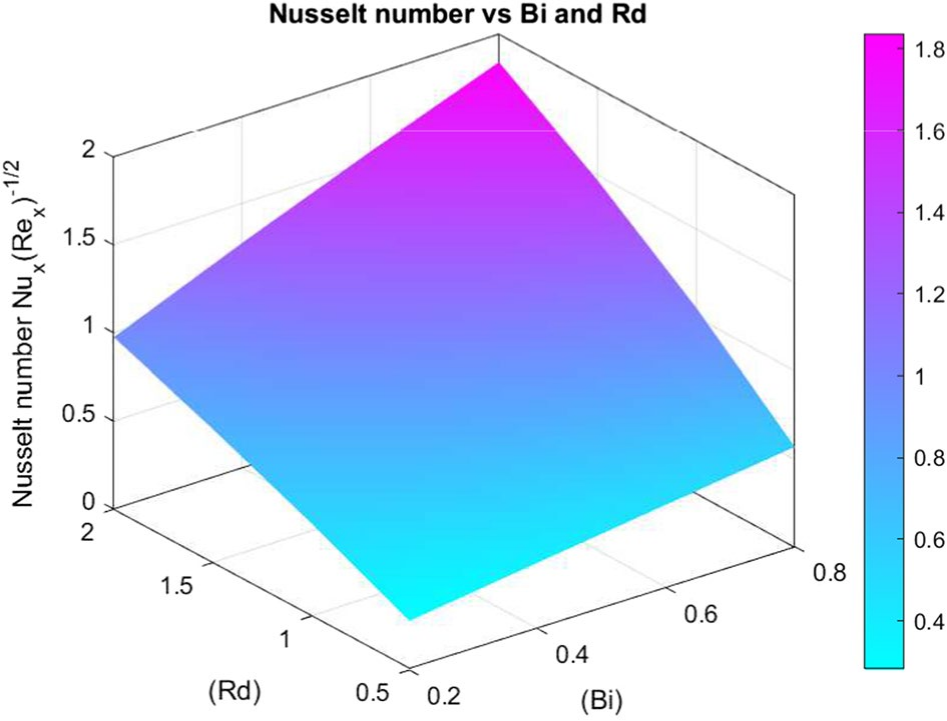
Heat transfer coefficient against *Bi* and *Rd*

**Fig. 28:**
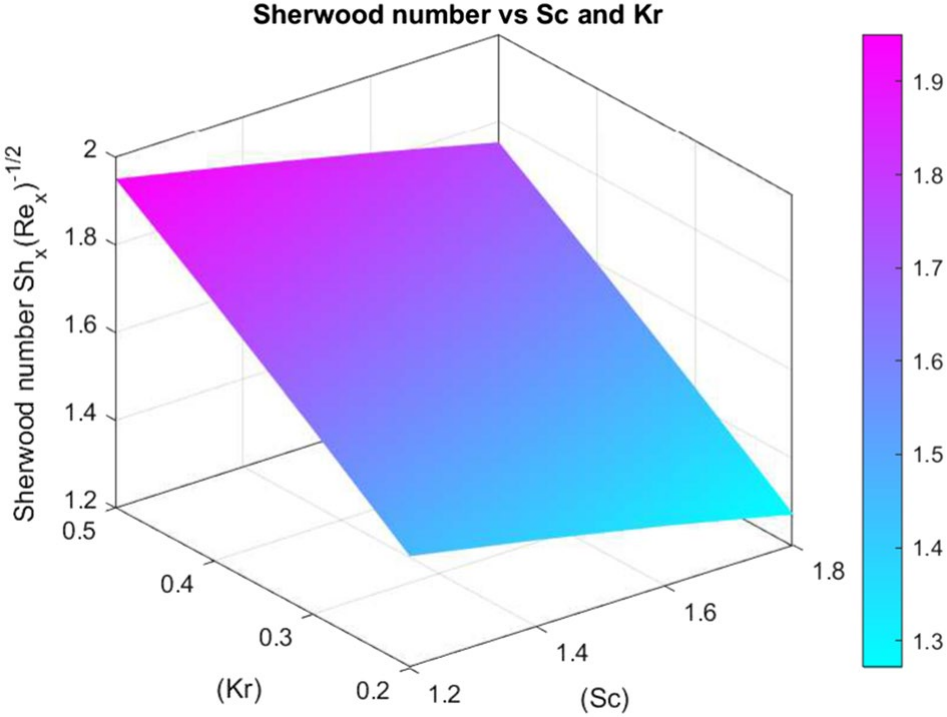
Mass transfer coefficient against $K_{r}$ and *Sc*

Figures 26, 27, and 28 show how significant dimensionless quantities changed in relation to pertinent physical factors in transport phenomena. The skin friction coefficient $\left( C_{f_x} Re_x^{1/2}\right)$ together with the magnetic parameter (*M*) and Weissenberg number (*We*) are shown in Fig. 26. By increasing either *M* or *We*, the skin friction coefficient decreases, indicating less shear stress at the wall as a consequence of higher surface tension and magnetic effects. The Nusselt number $\left( Nu_x Re_x^{-1/2}\right)$ is found in Fig. 27 as a function of the radiation parameter (*Rd*) and the Biot number (*Bi*). A higher *Bi* and *Rd* result in a larger Nusselt number, which indicates more heat transfer. Sherwood number $\left( Sh_x Re_x^{-1/2}\right)$ is shown against Schmidt number (*Sc*) and chemical reaction rate parameter (*Kr*) in Fig. 28. A higher Sherwood number indicates improved mass transfer and is caused by a rise in *Sc* or *Kr*. Plotting the effects of crucial dimensionless groups on heat transfer, skin friction, and mass transfer in fluid systems is a common practice.

## Multiple linear regression

**Fig. 29:**
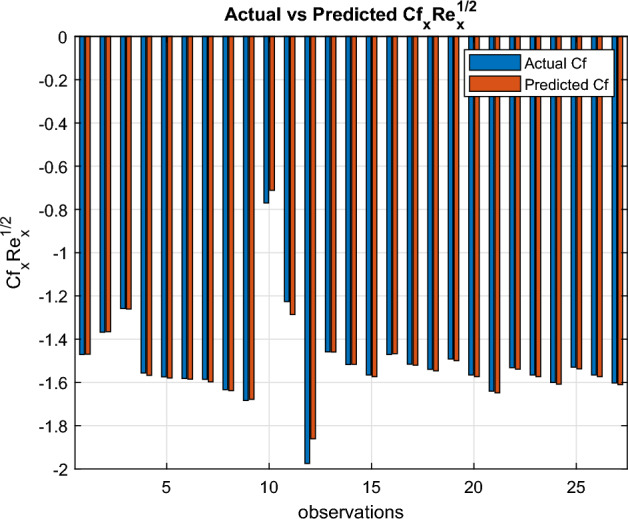
Actual versus estimated $Cf_xRe_x^{1/2}$

**Fig. 30:**
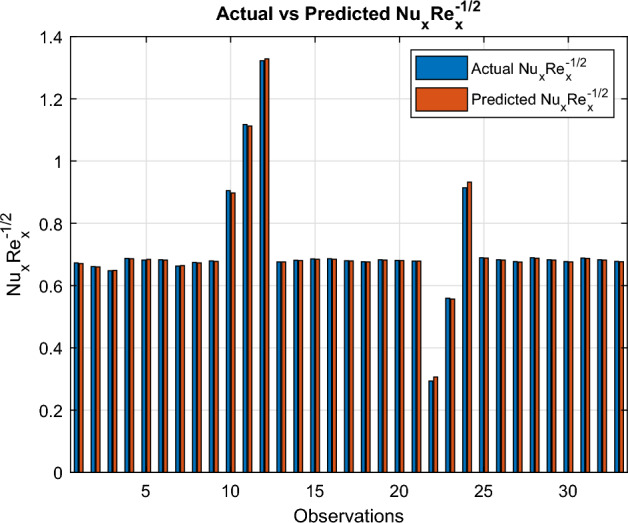
Actual versus estimated $Nu_xRe_x^{-1/2}$

**Fig. 31:**
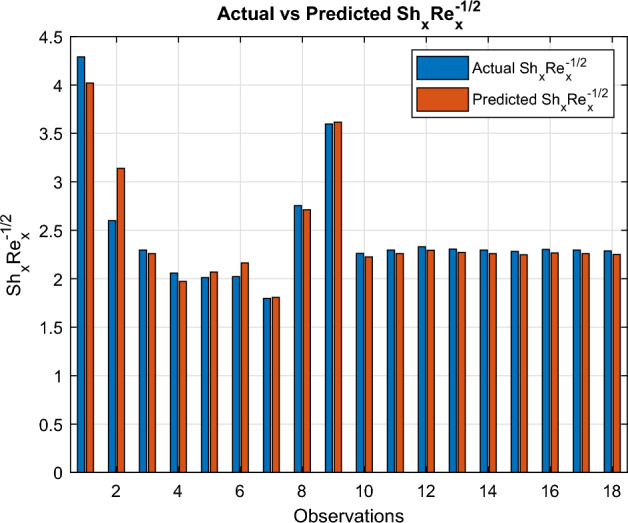
Actual versus estimated $Sh_xRe_x^{-1/2}$

**Table 6:** Coefficients of regression for Skin friction

	Coefficient	Standard error	*p*-value
Intercept	− 0.987601111	0.095885695	1.00247E−08
*M*	1.041581989	0.100895056	9.69072E−09
*n*	− 0.005838067	0.014619118	0.694611477
*We*	− 0.080348796	0.024145123	0.003984084
*S*	− 0.574519526	0.021689341	2.90688E−15
$\delta$	− 0.570114785	0.16536714	0.003074245
$E_1$	− 0.266666944	0.081862718	0.004636397
$\chi _1$	− 7.435000000	2.482246809	0.008136319
$\chi _2$	− 3.435000000	2.482246809	0.184312713
$\chi _3$	− 3.660000000	2.482246809	0.158633189

**Table 7:** Coefficients of regression for Nusselt Number

	Coefficient	Standard error	*p*-value
Intercept	− 0.149629311	− 3.116022530	0.005226375
*M*	− 0.110514012	0.015845982	6.90285E−07
$\delta$	− 0.021980383	0.026066194	0.408589918
$E_1$	0.044534708	0.012870937	0.002342563
*Rd*	0.430611483	0.003169196	2.08037E−32
*Pr*	0.008503164	0.007196055	0.250556465
*Ec*	− 0.028510898	0.023179474	0.232296743
*Q*	− 0.015019617	0.026066194	0.570597563
*Bi*	1.251718956	0.015036639	6.00191E−28
$\chi _1$	− 0.630000000	0.395073839	0.125733688
$\chi _2$	− 0.610000000	0.395073839	0.137521312
$\chi _3$	− 0.525000000	0.395073839	0.198151563

**Table 8:** Coefficients of regression for Sherwood number

	Coefficient	Standard error	*p*-value
Intercept	1.705674899	0.614658017	0.018064790
$\delta$	− 8.805239659	0.920864255	1.15454E−06
$K_r$	0.953900243	0.564663269	0.119266391
*Sc*	0.904258151	0.119924672	1.14152E−05
$\chi _1$	3.460000000	13.633767710	0.804345264
$\chi _2$	− 1.205000000	13.633767710	0.931160453
$\chi _3$	−0.805000000	13.633767710	0.953975615

**Table 9:** Regression Statistics for Skin friction, Nusselt number and Sherwood number

Regression statistics	$Cf_xRe_x^{1/2}$	$Nu_xRe_x^{-1/2}$	$Sh_xRe_x^{-1/2}$
Multiple *R*	0.989916342	0.999612005	0.965393795
R Square	0.979934364	0.99922416	0.93198518
Adjusted $R^2$	0.969311381	0.998817768	0.894886187
Standard Error	0.035104271	0.005587188	0.192810592
MAE (Mean Absolute Error)	0.013801360	0.002508742	0.084786555
MAPE (Mean Absolute Percentage Error)	1.009792832	0.396954110	3.299365220
MSE (Mean Squared Error)	0.000775899	0.000019865	0.022718620
RMSE (Root Mean Squared Error)	0.027854960	0.004457034	0.150726973

A statistical technique called multiple linear regression models the connection between a number of independent factors and a dependent variable using a linear equation:

$$\begin{aligned} y = p_0 + p_1x_1 + p_2x_2 + \cdots + p_nx_n \end{aligned}$$

where:$y$ denotes the response (dependent) variable, $x_1, x_2, \ldots , x_n$ are the explanatory (independent) variables, $p_0$ represents the intercept of the model, $p_i$ are the regression coefficients linked to each $x_i$, with $i = 1, 2, 3, \ldots , n$

An independent variable $x_i$ is considered to exert a statistically meaningful influence on $y$ when the corresponding p-value for $p_i$ is below the threshold of 0.05.

$$\begin{aligned} \begin{aligned} Cf_{\text {est}}&= l_0 + l_1 M + l_2 n + l_3 We + l_4 S \\&\quad + l_5 \delta + l_6 E_1 + l_7 \chi _1 + l_8 \chi _2 + l_9 \chi _3 \\ Nu_{est}&= p_0+ p_1 M+ p_2 \delta + p_3 E_1+p_4 Rd + p_5 Pr+ p_6 Ec\\&\quad + p_7 Q+ p_8 We +p_{9} Bi + p_{10}\chi _1+ p_{11} \chi _2 + p_{12} \chi _3\\ Sh_{est}&= q_0+ q_1 \delta + q_2 K_r + q_3 Sc + q_4 \chi _1 + q_5 \chi _2+ q_6 \chi _3 \end{aligned} \end{aligned}$$

In Microsoft Excel, regression coefficients are computed. In Figs. 29, 30 and 31, the actual and projected values of the Sherwood number, Nusselt number and skin friction coefficient are compared. The matching regression models are provided below:

$$\begin{aligned} \begin{aligned} Cf_{est}&= -0.987601111 + 1.041581989 M -0.005838067 n \\&\quad -0.080348796 We -0.574519526 S -0.570114785 \delta \\&\quad -0.266666944 E_1 -7.435 \chi _1 -3.435 \chi _2 -3.66 \chi _3\\ Nu_{est}&= -0.149629311 -0.110514012 M -0.021980383 \delta + 0.044534708 E_1\\&+0.430611483 Rd + 0.008503164 Pr-0.028510898 Ec\\&\quad -0.015019617 Q+ 1.251718956 Bi -0.63 \chi _1 -0.61 \chi _2 -0.525 \chi _3 \\ Sh_{est}&= 1.705674899 -8.805239659 \delta + 0.953900243 K_r \\&\quad + 0.904258151 Sc + 3.46 \chi _1 -1.205 \chi _2 -0.805 \chi _3 \end{aligned} \end{aligned}$$

The regression coefficient Tables 6, 7, and 8 show the effects of various physical parameters on skin friction, Nusselt number, and Sherwood number. Low *p*-value parameters significantly affect the corresponding transport phenomena. The regression performance indicators for the skin-friction coefficient ($Cf_x Re_x^{1/2}$), Nusselt number ($Nu_x Re_x^{-1/2}$), and Sherwood number ($Sh_x Re_x^{-1/2}$) are compiled in Table 9. Every model shows good predictive power, with $R^2$ values over 0.93. With the lowest MAE (0.0025), the greatest $R^2$ (0.9992), and the MAPE ($0.39\%$), the Nusselt number model exhibits the best performance. The Sherwood number model has a somewhat larger prediction error but a reasonable $R^2$ of 0.9319 and acceptable MAE and RMSE values. These results demonstrate how well the regression models capture the transport properties of nanofluid flow. In bar graphs (29, 30, and 31), the three dimensionless numbers $Cf_x Re_x^{1/2}$, $Nu_x Re_x^{-1/2}$, and $Sh_x Re_x^{-1/2}$ across many observations are compared to their actual and expected values. Actual data is represented by blue bars, while anticipated values are shown by orange bars. The dependability of the models for predicting heat transfer, mass transfer, and skin friction is confirmed by the strong agreement between the actual and anticipated values in all three images, which shows how well the models reproduce the underlying transport behaviour.

## Conclusion

This study examined the unsteady flow and thermal characteristics of a Carreau ternary hybrid nanofluid ($Cu + Al_2O_3 + TiO_2$ in a CMC–water base fluid) over a stretching sheet, considering Ohmic dissipation, electromagnetic forces, thermal radiation, and heat source/sink effects. We used similarity transformations to simplify the governing equations and then solved them numerically. The findings indicate that:

- Ternary hybrid nanofluids substantially outperform mono and binary nanofluids in enhancing both velocity and heat transfer, particularly at high Weissenberg and magnetic parameters.
- Radiation and viscous dissipation further increase Nusselt numbers, signifying improved heat transfer performance.
- Entropy generation rises with *Br*, *We*, *M*, and radiation effects, indicating a trade-off between enhanced heat transport and irreversibility.
- Bejan number analysis reveals that thermal irreversibility dominates under strong radiative and electric effects, while frictional irreversibility becomes more significant at higher magnetic parameters.

Regression analysis provided strong quantitative validation, with correlations for skin friction, Nusselt, and Sherwood numbers showing excellent agreement with established literature and commercial CFD benchmarks (e.g., $R^2 > 0.93$ and MAPE < 3.5%), thus confirming the model’s reliability. The present approach offers high prediction accuracy while maintaining a lower computational cost compared to comprehensive CFD simulations, rendering it suitable for engineering design and optimisation.

The findings demonstrate the engineering significance of ternary hybrid nanofluids in advanced thermal management systems. Some such uses are:

- **Polymer and extrusion processes**, where precise temperature control is crucial for product quality.
- **Microfluidics and biomedical devices**, benefiting from improved thermal regulation at microscale.
- **Electronic cooling and high-heat-flux components**, where enhanced conductivity and convective performance reduce overheating risks.
- **Renewable energy systems**, such as solar collectors and thermal storage units, where nanofluids can maximize energy capture and transfer.
- **Aerospace and automotive thermal systems**, offering lightweight, efficient heat dissipation under extreme conditions.

This analysis provides useful insights for polymer sheet extrusion, thermal coating, microelectronic cooling, and heat exchanger systems that use electrically conductive nanofluids. Regression-based correlations allow engineers to predict heat and mass transfer rates efficiently. This suggests that the proposed ternary hybrid nanofluid is suitable for practical use in thermal management and coating technologies.

### Limitations and future work

This analysis focusses on two-dimensional, laminar boundary-layer flow characterised by uniform nanoparticle dispersion and unsteady-state conditions, while excluding agglomeration and turbulence effects. Future extensions will encompass turbulent and transient regimes, nanoparticle clustering, and complex geometries. Additionally, hybrid approaches that integrate machine learning, ANN, Response Surface Method(RSM) with CFD will be explored to improve prediction, optimisation, and real-time control in industrial thermal systems.

## Data Availability

Included in the paper or Supplementary Information (for raw data, not summary data such as means and variances).

## List of symbols

$E_0$: Electric field coefficient
$E_1$: Electric field variable
*E*: Applied electric field
*B*: Applied magnetic field
$B_0$: Uniform magnetic field intensity (Tesla)
$$\tilde{T}_w$$: Surface temperature (K)
$\tilde{C}_w$: Surface concentration (kg m^−3^)
$\tilde{T}_\infty$: Temperature in the far field
$\tilde{C}_\infty$: Concentration in the far field
$c_p$: Heat capacity (J kg^−1^ K^−1^)
$\tilde{k}$: Mean absorption coefficient
*k*: Thermal conductivity (W m^−1^ K^−1^)
$$\Gamma $$: Material parameter
*n*: Power-law index
$h_f$: Heat transfer coefficient (W m^−2^ K^−1^)
*Q*: Heat Source parameter
$q_r$: Radiative heat energy flux (W m^−2^)
$$\tilde{u}_w$$, $$\tilde{v}_w$$: velocity components
*S*: Suction parameter

## Greek symbols

$$\nu $$: Kinematic viscosity (m^2^ s^−1^)
$$\delta $$: Unsteadiness parameter
$\mu$: Dynamic viscosity (kg m^−1^ s^−1^)
$\rho$: Mass density (kg m^−3^)
$$\sigma $$: Electrical conductivity (S m^−1^)
$$\theta $$: Dimensionless temperature
$$\phi $$: Dimensionless concentration
$$\eta $$: Similarity variable

## Subscripts

*thnf*: Ternary hybrid nanofluid
*hnf*: Hybrid nanofluid
*nf*: Nanofluid
*f*: Base fluid
1: First nanoparticle
2: Second nanoparticle
3: Third nanoparticle

## References

[1] Choi SU, Eastman JA. Enhancing thermal conductivity of fluids with nanoparticles. Argonne National Lab. (ANL), Argonne, IL, 10; 1995

[2] Bi SS, Shi L, Zhang LL. Application of nanoparticles in domestic refrigerators. Appl Therm Eng. 2008;28(14):1834–43.

[3] Carreau PJ. Rheological equations from molecular network theories. Trans Soc Rheol. 1972;16(1):99–127.

[4] Khan M, Hashim. Boundary layer flow and heat transfer to carreau fluid over a nonlinear stretching sheet. AIP Adv. 2015;5(10):107203.

[5] Tian J, Rehman S, Saqib M, Shah AG, AlAbdulaal T. Entropy generation and heat transport performance of a partially ionized viscoelastic tri-hybrid nanofluid flow over a convectively heated cylinder. Case Stud Therm Eng. 2024;60:104623.

[6] Zafar S, Zaib A, Ali F, Faizan M, Khan U, Sherif E-SM, et al. Dynamics of active and passive control on bioconvection carreau nanofluid with thermal radiation and cattaneo-christov double-diffusion effects. J Radiat Res Appl Sci. 2025;18(2):101336.

[7] Zafar S, Zaib A, Faizan M, Shah NA, Ali F, Yook S-J. Bioconvection flow of prandtl nanomaterial due to stretched cylinder enclosed through darcy forchheimer flow with triple stratification. Alex Eng J. 2025;116:188–201.

[8] Bachok N, Ishak A, Pop I. Unsteady boundary-layer flow and heat transfer of a nanofluid over a permeable stretching/shrinking sheet. Int J Heat Mass Transf. 2012;55(7):2102–9.

[9] Shah NA, Ali F, Faizan M, Zafar SS. Theoretical exploration of bioconvection magneto flow of micropolar nanomaterial off-centered stagnation point framed by rotating disk. Adv Theory Simul. 2025;8(6):2401345.

[10] Nandi S, Kumbhakar B. Navier’s slip effect on carreau nanouid flow past a convectively heated wedge in the presence of nonlinear thermal radiation and magnetic field. Int Commun Heat Mass Transfer. 2020;118:104813.

[11] Rehman S, Bouzgarrou S, Houcine Dhaou HM, Boujelbene M. Darcy-Forchheimer flow of bioconvective nanofluid over a nonaligned stretching surface with slip effects. Mater Today Commun. 2023;37:107444.

[12] Rehman S, khan Z, Ali H, Riaz U, Albouchi F. Role of nanomaterial on irreversibility and heat transport due to stretching surface driven blood flow in the view of buongiorno and tiwari-das models. Ain Shams Eng J. 2024;15(6):102747.

[13] Kaswan P, Kumar M, Kumari M, Mandal G. Entropy minimization of mhd hybrid nanofluid flow between two concentric cylinders with nonlinear thermal radiation. Hybrid Adv. 2025;11:100533.

[14] Kumar M, Kaswan P, Kumari M. Entropy generation analysis of microrotating Casson’s nanofluid with Darcy-Forchheimer porous media using a neural computing based on levenberg-marquardt algorithm. Int J Numer Methods Heat Fluid Flow. 2024;34:2285–320.

[15] Agrawal R, Kaswan P. Entropy generation minimization of ag-fe_3o_4/water-ethylene glycol squeezed hybrid nanofluid flow between parallel disks. Int J Numer Methods Heat Fluid Flow. 2022;33:65–9.

[16] Kumar M, Kaswan P, Kumari M, Ahmad H, Askar S. Cattano Christov double diffusion model for third grade nanofluid flow over a stretching riga plate with entropy generation analysis. Heliyon. 2024;10(10):e30188. 38803878 10.1016/j.heliyon.2024.e30188 PMC11128471

[17] Ahmed MF, Zaib A, Ali F, Khan U, Zafar SS. Thermal radiation of Walter-b magneto bioconvection nanofluid due to the stretching surface under convective condition and heat source/sink: a semi-analytical technique for the stagnation point. J Radiat Res Appl Sci. 2025;18(1):101291.

[18] Shah NA, Ali F, Yook S-J, Faizan M, Zafar S, Sidi MO. Dynamics of chemical reactive on magneto hybrid nanomaterial with heat radiation due to porous exponential plate: Laplace transform technique for the heat and mass. J Radiat Res Appl Sci. 2025;18(1):101295.

[19] Jubair S, Ali B, Kumar A, Ansari MA. Numerical simulation of nonlinear flow and energy dynamics through spinning flow of casson hybrid nanofluid past an extending surface. Part Sci Technol. 2025;43(4):667–79.

[20] Shahzad F, Jamshed W, Sathyanarayanan SUD, Aissa A, Madheshwaran P, Mourad A. Thermal analysis on darcy-forchheimer swirling casson hybrid nanofluid flow inside parallel plates in parabolic trough solar collector: an application to solar aircraft. Int J Energy Res. 2021;45(15):20812–34.

[21] Rehman S, Alfaleh A, Afef K, Hashim, Shah, SIA. Onset about isothermal flow of Carreau liquid over converging channel with Cattaneo-Christov heat and mass fluxes. Heliyon. 2023;9(5):e15710. 37215786 10.1016/j.heliyon.2023.e15710 PMC10195909

[22] Ouni M, Ladhar LM, Omri M, Jamshed W, Eid MR. Solar water-pump thermal analysis utilizing copper–gold/engine oil hybrid nanofluid flowing in parabolic trough solar collector: thermal case study. Case Stud Therm Eng. 2022;30:101756.

[23] Mkhatshwa MP, Khumalo M. Irreversibility scrutinization on emhd Darcy–Forchheimer slip flow of carreau hybrid nanofluid through a stretchable surface in porous medium with temperature-variant properties. Heat Transf. 2023;52(1):395–429.

[24] Johnson AB, Olajuwon BI. Impact of radiation and heat generation/absorption in a Walters’ b fluid through a porous medium with thermal and thermo diffusion in the presence of chemical reaction. Int J Model Simul. 2023;43(2):87–100.

[25] Jamshed W, Safdar SUDSR, Redouane F, Nisar KS. Comprehensive analysis on copper-iron (ii, iii)/oxide-engine oil casson nanofluid flowing and thermal features in parabolic trough solar collector. J Taibah Univ Sci. 2021;15(1):619–36.

[26] Jamshed W, Eid MR, Azeany Mohd Nasir, K. S. Nisar NA, Aziz A, Shahzad F, Shukla A. Thermal examination of renewable solar energy in parabolic trough solar collector utilizing maxwell nanofluid: a noble case study. Case Stud Therm Eng. 2021;27:101258.

[27] Jamshed W, Nisar KS. Computational single-phase comparative study of a Williamson nanofluid in a parabolic trough solar collector via the Keller box method. Int J Energy Res. 2021;45(7):10696–718.

[28] Ali B, Jubair S, Siddiqui MIH. Numerical simulation of hybrid nanofluid flow consisting of polymer–cnt matrix nanocomposites subject to Lorentz force and heat source/sink across coaxial cylinders. Mod Phys Lett B. 2025;39(01):2450386.

[29] Ali B, Zhou Y-T, Jubair S, Tariq MH, Kumar A, Haque Siddiqui MI. Entropy optimization and prandtl-eyring non-newtonian fluid flow with second-order slip conditions past a curved riga sheet; numerical simulation. Int J Therm Sci. 2025;214:109916.

[30] Jubair S, Ali B, Rafique K, Mahmood Z, Emam W. Numerical simulation of hybrid nanofluid flow with homogeneous and heterogeneous chemical reaction across an inclined permeable cylinder/plate. Energy Explor Exploit. 2024;42(6):2270–88.

[31] Khedher NB, Rehman S, Alqahtani S, Hashim SA. Comparative study of entropy distribution for generalized fluid between an inclined channel in the perspective of classical and non-fourier’s law. Eng Sci Technol Int J. 2023;45:101471.

[32] Chakraborty R, Dey R, Chakraborty S. Thermal characteristics of electromagnetohydrodynamic flows in narrow channels with viscous dissipation and joule heating under constant wall heat flux. Int J Heat Mass Transf. 2013;67:1151–62.

[33] Rehman S, Shamshad M, Nasr S, Abdullaev S. Thermal characteristics of mhd cofe2o4/ water nanofluids flow past a stretching/shrinking wedge in the view of cattaneo-christov heat flux. Case Stud Therm Eng. 2024;56:104225.

[34] Alkathiri AA, Jamshed W, Uma Devi S S, Eid MR, Bouazizi ML. Galerkin finite element inspection of thermal distribution of renewable solar energy in presence of binary nanofluid in parabolic trough solar collector. Alex Eng J. 2022;61(12):11063–76.

[35] Ali F, Kamal M, Faizan M, Zafar SS. Mixed convective magneto flow of nanofluid for the sutterby fluid containing micro-sized selfpropelled microorganisms with chemical reaction: Keller box analysis. Phys Scr. 2024;99:095202.

[36] Nandi S, Vajravelu K. Analysis of entropy generation in carreau ternary hybrid nanofluid flow over a stretching sheet. Numer Heat Transf Part A Appl. 2024;85(19):3209–33.

[37] Makhdoum BM, Mahmood Z, Fadhl BM, Aldhabani MS, Khan U, Eldin SM. Significance of entropy generation and nanoparticle aggregation on stagnation point flow of nanofluid over stretching sheet with inclined lorentz force. Arab J Chem. 2023;16(6):104787.

[38] Rafique K, Mahmood Z, Saleem S, Eldin SM, Khan U. Impact of nanoparticle shape on entropy production of nanofluid over permeable mhd stretching sheet at quadratic velocity and viscous dissipation. Case Stud Therm Eng. 2023;45:102992.

[39] Maiti H, Khan AY, Mondal S, Nandy SK. Scrutinization of unsteady mhd fluid flow and entropy generation: Hybrid nanofluid model. J Comput Math Data Sci. 2023;6:100074.

[40] Boujelbene M, Rehman S, Jazaa Y, Hashim, Dhaou MH. Thermodynamics of hydromagnetic boundary layer flow of prandtl nanofluid past a heated stretching cylindrical surface with interface slip. J Taiwan Inst Chem Eng. 2024;155:105310.

[41] AlAbdulaal T, Rehman S, Rauf S, Albouchi F, Abduvalieva D. Optimization of entropy and heat transfer in a magnetohydrodynamic marangoni convection flow of biviscosity bingham hybrid nanofluid through convergent channel. Case Stud Therm Eng. 2024;61:105019.

[42] Shahzad F, Jamshed W, Safdar R, Hussain SM, Nasir NAAM, Dhange M, et al. Thermal analysis characterisation of solar-powered ship using oldroyd hybrid nanofluids in parabolic trough solar collector: An optimal thermal application. Nanotechnol Rev. 2022;11(1):2015–37.

[43] Jamshed W, Shahzad F, Safdar R, Sajid T, Eid MR, Nisar KS. Implementing renewable solar energy in presence of maxwell nanofluid in parabolic trough solar collector: a computational study. Waves Random Complex Media. 2024;34(5):4320–51.

[44] Arafat H, Ali F, Shah NA, Sidi MO, Yook S-J. Theoretical analysis of activation energy of Darcy-Forchheimer fractional bioconvection flow of nanofluid due to a rotating disk with entropy generation. Fractals. 2025;33(10):2540247.

[45] Priyadharshini P, Divya P, Vanitha Archana M. Impact of multiple linear regression strategies on unsteady thin film flow of Al2O3 nanofluid over a stretching sheet. J Nanofluids. 2024;13(5):1080–95.

[46] Asiri F, Rehman S, Drissi N. Sensitivity analysis of Cattaneo–Christov heat and mass flux model effects in Stefan blowing flow of ferromagnetic nanofluid: numerical simulations. Results Chem. 2025;16:102490.

[47] Blessy TG, Kumar BR. Simulation and non-similar analysis of magnetized swcnt-mwcnt hybrid nanofluid flow in porous media using darcy-forchheimer-brinkman model. Case Stud Therm Eng. 2024;64:105421.

[48] Ayub A, Shah SZH, Sabir Z, Rao NS, Sadat R, Ali MR. Spectral relaxation approach and velocity slip stagnation point flow of inclined magnetized cross-nanofluid with a quadratic multiple regression model. Waves Random Complex Media. 2025;35(2):3131–55.

[49] Alimi F, Rehman S, Bouzidi M, Asiri F, Saidani T, Tirth V. Numerical and artificial neural network framework for predicating mhd radiative flow and heat transfer of hybrid nanofluid with cattaneo-christov theory. Case Stud Therm Eng. 2025;72:106311.

[50] Jubair S, Yang J, Ali B, Bin-Mohsin B, Khalifa HAE-W. Analyzing the impact of non-Newtonian nanofluid flow on pollutant discharge concentration in wastewater management using an artificial computing approach. Appl Water Sci. 2024;15(1):8.

[51] Ullah H, Awan FJ, Maqbool K, Lu D. Computational analysis of carreau fluid flow in presence with buoyancy force, viscous dissipation, and chemical reaction. J Mech. 2024;40:377–83.

[52] Gangadhar K, Sangeetha Rani M, Subbarao K, Wakif A. Analysis of carreau triple nanoparticle suspension on flow over an elongating surface with ohmic dissipation. Eur Phys J Plus. 2023;138(11):1035.

[53] Sindhu T, Jagadeeshkumar K, Reddy AS. Unsteady mhd flow and entropy generation in carreau tetra-hybrid nanofluid over an inclined stretching surface with cattaneo-christov heat flux’’. J Therm Anal Calorim. 2025;150:16771.

[54] Ramzan M, Ali F, Akkurt N, Saeed A, Kumam P, Galal AM. Computational assesment of carreau ternary hybrid nanofluid influenced by mhd flow for entropy generation. J Magn Magn Mater. 2023;567:170353.

[55] Brinkman H. C. The viscosity of concentrated suspensions and solutions. J Chem Phys. 1952;20:571–571.

[56] Pak BC, Cho YI. Hydrodynamic and heat transfer study of dispersed fluids with submicron metallic oxide particles. Exp Heat Transf. 1998;11(2):151–70.

[57] Maxwell JC. A treatise on electricity and magnetism, vol. 1. New York: Oxford University Press; 1873.

[58] Mukhopadhyay S, Bhattacharyya K. Unsteady flow of a maxwell fluid over a stretching surface in presence of chemical reaction. J Egyptian Math Soc. 2012;20(3):229–34.

[59] Chamkha AJ, Aly AM, Mansour MA. Similarity solution for unsteady heat and mass transfer from a stretching surface embedded in a porous medium with suction/injection and chemical reaction effects. Chem Eng Commun. 2010;197(6):846–58.

[60] Shafie S, Mahmood T, Pop I. Similarity solutions for the unsteady boundary layer flow and heat transfer due to a stretching sheet. Int J Appl Mech Eng. 2006;11:01.

